# MRI-based radiomics for predicting pathological complete response after neoadjuvant chemoradiotherapy in locally advanced rectal cancer: a systematic review and meta-analysis

**DOI:** 10.3389/fonc.2025.1550838

**Published:** 2025-03-10

**Authors:** Zhongfan Liao, Dashuang Luo, Xiaoyan Tang, Fasheng Huang, Xuhui Zhang

**Affiliations:** Department of Oncology, Sichuan Provincial People’s Hospital, University of Electronic Science and Technology of China, Chengdu, China

**Keywords:** magnetic resonance imaging, radiomics, rectal neoplasms, neoadjuvant chemoradiotherapy, meta-analysis

## Abstract

**Purpose:**

To evaluate the value of MRI-based radiomics for predicting pathological complete response (pCR) after neoadjuvant chemoradiotherapy (NCRT) in patients with locally advanced rectal cancer (LARC) through a systematic review and meta-analysis.

**Methods:**

A systematic literature search was conducted in PubMed, Embase, Proquest, Cochrane Library, and Web of Science databases, covering studies up to July 1st, 2024, on the diagnostic accuracy of MRI radiomics for predicting pCR in LARC patients following NCRT. Two researchers independently evaluated and selected studies using the Quality Assessment of Diagnostic Accuracy Studies 2 (QUADAS-2) tool and the Radiomics Quality Score (RQS) tool. A random-effects model was employed to calculate the pooled sensitivity, specificity, and diagnostic odds ratio (DOR) for MRI radiomics in predicting pCR. Meta-regression and subgroup analyses were performed to explore potential sources of heterogeneity. Statistical analyses were performed using RevMan 5.4, Stata 17.0, and Meta-Disc 1.4.

**Results:**

A total of 35 studies involving 9,696 LARC patients were included in this meta-analysis. The average RQS score of the included studies was 13.91 (range 9.00-24.00), accounting for 38.64% of the total score. According to QUADAS-2, there were risks of bias in patient selection and flow and timing domain, though the overall quality of the studies was acceptable. MRI-based radiomics showed no significant threshold effect in predicting pCR (Spearman correlation coefficient=0.119, P=0.498) but exhibited high heterogeneity (I^2^≥50%). The pooled sensitivity, specificity, positive likelihood ratio, negative likelihood ratio and DOR were 0.83, 0.82, 5.1, 0.23 and 27.22 respectively, with an area under the summary receiver operating characteristic (sROC) curve of 0.91. According to joint model analysis, publication year, country, multi-magnetic field strength, multi-MRI sequence, ROI structure, contour consistency, feature extraction software, and feature quantity after feature dimensionality reduction were potential sources of heterogeneity. Deeks’ funnel plot suggested no significant publication bias (P=0.69).

**Conclusions:**

MRI-based radiomics demonstrates high efficacy for predicting pCR in LARC patients following NCRT, holding significant promise for informing clinical decision-making processes and advancing individualized treatment in rectal cancer patients.

**Systematic review registration:**

https://www.crd.york.ac.uk/prospero/, identifier CRD42024611733.

## Introduction

The standard treatment for locally advanced rectal cancer (LARC) is neoadjuvant chemoradiotherapy (NCRT) combined with total mesorectal excision (TME). Study reported that approximately 15-27% of LARC patients exhibited no residual viable tumor cells upon pathological examination after NCRT, indicating a pathological complete response (pCR) (1). Some studies suggested a “watch and wait” strategy for patients achieving pCR, noting no significant differences in distant metastasis rate, disease-free survival, or overall survival compared to those who undergo surgery (2, 3). Therefore, some studies recommended preoperative evaluation for LARC patients after NCRT, allowing for a “watch and wait” approach for those who achieve pCR. This strategy can help avoid permanent stoma formation and postoperative complications, thereby improving patients’ quality of life (4, 5). Consequently, accurate preoperative prediction of pCR following NCRT can impact on clinical decision-making and enhance quality of life.

Imaging modalities are the mainstay of preoperative prediction of pCR. However, studies have shown that conventional imaging methods did not achieve ideal predictive results (6, 7). Currently, most studies focused on evaluating the efficacy of NCRT through radiomics, a machine learning approach that enables high-throughput extraction and quantitative analysis of numerous imaging features from radiographic images. Compared with the subjective analysis of conventional imaging, the advantage of radiomics lies in the ability to quantitatively analyze, identify, and reveal deep features within images that are difficult to discern with the naked eye, effectively overcoming the limitations of subjectivity in manual image recognition (8). The basic workflow of radiomics can be divided into five main steps: image acquisition, image segmentation, feature extraction and quantification, feature dimensionality reduction and selection, and model construction.

Magnetic resonance imaging (MRI) offers high-resolution imaging of soft tissues, enabling clear visualization of structures such as cancer nests and fibrosis in rectal cancer following NCRT. Numerous studies have employed MRI-based radiomics to predict whether LARC patients achieve pCR following NCRT. However, discrepancies remained among study outcomes, and there is a lack of latest research providing a comprehensive systematic review and meta-analysis of MRI-based radiomics for the prediction of pCR (9). This study aims to explore the predictive value of MRI-based radiomics for pCR by screening and evaluating relevant studies, thereby providing evidence-based guidance for clinical decision-making and prognostic management.

## Materials and methods

### Study design

The Preferred Reporting Items for Systematic Reviews and Meta-Analyses of Diagnostic Test Accuracy Studies (PRISMA-DTA) guidelines were followed for conducting this systematic review (10). There was no systematic review relevant to the topic of this study that was identified in the Cochrane library.

### Population, intervention, comparison, outcome

- Population: LARC patients undergoing preoperative MRI examination, either before or after NCRT, with TRG confirmed by histopathology after TME.
- Intervention: Radiomics analysis was performed with preoperative MRI images of tumors, classifying NCRT response as pCR versus non-pCR.
- Comparison: The predictive performance of MRI-based radiomics in comparison to the pathological gold standard was evaluated.
- Outcome: The efficacy of MRI-based radiomics to predict pCR after NCRT in patients with LARC was evaluated through a diagnostic accuracy study design (e.g., ROC curve analysis).

### Search strategy

A combination of MeSH terms and free text words was used for an online search in the PubMed, Embase, Proquest, Cochrane Library, and Web of Science databases, covering the period from database inception to July 1, 2024. Detailed search terms and strategies are provided in **Supplementary Table S1**. To avoid duplication and prevent omissions, the retrieved documents were cross-checked, and citation tracking was conducted.

### Inclusion and exclusion criteria

Inclusion criteria: (1) studies involving diagnostic accuracy MRI radiomics; (2) all subjects were required to undergo preoperative rectal MRI; (3) the experimental group consisted of LARC patients achieving pCR after NCRT, while the control group included LARC patients with non-pCR after NCRT; (4) the gold standard for the diagnosis of pCR is postoperative histopathological biopsy; (5) sufficient data to directly extract or indirectly calculate the numbers of true positives (TP), false positives (FP), true negatives (TN), and false negatives (FN) cases.

Exclusion criteria: (1) non-diagnostic studies, including reviews, case reports, experimental studies, or conference abstracts; (2) duplicate publications; (3) studies with inaccessible full text or incomplete data; (4) studies with a sample size of 20 or fewer cases; (5) studies with a RQS score below 5.

### Literature screening and data extraction

Initially, two researchers (each with over three years of experience in radiomics analysis) independently screened the titles and abstracts of the retrieved articles, excluding irrelevant studies. Each excluded study was re-evaluated by the different researcher. Subsequently, relevant studies were included in this systematic review after a careful and thorough full-text review.

Data extraction was conducted for the included studies, including general data and detailed radiomics data. General data included: (1) first author, (2) publication year, (3) publication country, (4) study type, (5) sample size, (6) average age, (7) chemoradiotherapy regimen, (8) imaging acquisition timing, (9) MRI field strength, sequence, and slice thickness, and (10) 2x2 table (TP, FP, TN, FN). Detailed radiomics data included: (1) ROI delineation software, method, and structure, (2) ROI contour consistency assessment, (3) feature extraction software, (4) feature types, (5) feature quantity after feature dimensionality reduction, (6) normalization methods, (7) feature dimensionality reduction and selection methods, (8) modeling algorithms, (9) model validations, and (10) optimal predictive model with AUC. In case of discrepancies, the decision will be referred to a senior researcher (with over ten years of experience in systematic review) for adjudicating, or consensus will be reached through consultation. When multiple modeling algorithms were applied to the same sample in a study, the model with the best classification performance was considered the optimal predictive model. If the optimal predictive model in a study was developed using data beyond MRI alone (such as PET-CT, ultrasound, pathological features, or clinical features), only data based on MRI and clinical features were extracted.

### Methodological quality assessment

#### QUADAS-2

The quality of the included studies was evaluated using the Quality Assessment of Diagnostic Accuracy Studies 2 (QUADAS-2) tool, specifically including patient selection, index test, reference standard, and flow and timing (11). If any item within a section was answered as “No”, it was rated as “high risk of bias”, which indicated potential methodological flaws that could impact diagnostic accuracy. If all items in a section were answered as “Yes”, it was rated as “low risk of bias”, which indicated that the study followed a sound methodological design unlikely to introduce bias. If the content reported was difficult to evaluate, it was rated as “unclear”, indicating insufficient information to judge the risk of bias. **Supplementary Table S2** provided detailed scoring criteria for each QUADAS-2 item.

#### RQS

The rigor and reproducibility of the included studies were assessed using the Radiomics Quality Score (RQS) proposed by Lambin (12). The RQS provides a meticulous assessment of 16 aspects across five key steps in the radiomics analysis, including data selection, medical imaging, feature extraction, exploratory analysis, and modeling. RQS both rewards and penalizes the methodology and statistical analysis of research, thereby promoting best scientific practices. The total score is 36, representing a 100% RQS score. Two researchers independently assessed the RQS score of each study, with disagreements resolved through consensus. **Supplementary Table S3** showed detailed scoring criteria for each RQS item.

### Statistical analysis

Review Manager (Cochrane; version 5.4) software was used to perform methodological quality assessment with built-in QUADAS-2 tool and to plot the risk of bias and applicability graphs. Meta-Disc (XI Cochrane Colloquium, Barcelona, Spain; version 1.4) software was used to calculate the Spearman correlation coefficient between the logit of sensitivity and the logit of 1-specificity. Summary receiver operator characteristic (sROC) curve was plotted to assess threshold effects in the pooled results. A Spearman correlation coefficient with P < 0.05 or a “shoulder arm” shape in the sROC curve scatters distribution indicated a threshold effect. If there was no threshold effect, heterogeneity among studies was analyzed using the inconsistency index (I^2^) and Cochrane Q test. When I^2^ > 50% and P < 0.05, which indicated the presence of heterogeneity, a random effects model was applied to analyze sampling error and variance across studies, and potential sources of heterogeneity should be explored. When I^2^ < 50% or P > 0.05, which indicated lack of heterogeneity, a fixed effects model should be used, which assumed that all effect sizes come from a single population and differences are due to chance.

In this study, a random effects model was applied, and the pooled effect sizes were calculated using Meta-Disc software, including sensitivity, specificity, positive likelihood ratio, negative likelihood ratio, diagnostic odds ratio (DOR), and area under the sROC curve (AUC). All analyses were conducted on the validation or test cohorts. Forest plots and sROC curves were plotted to visually display pooled effect sizes results. To further investigate the sources of heterogeneity, the MIDAS module in Stata (Stata Corporation, College Station, TX, USA; version 17.0) was used for meta-regression and subgroup analyses, which incorporated covariates into a dichotomy model to assess the impact of various factors on the predictive performance. The following factors were considered as potential sources of heterogeneity: publication year (≥2021 vs. <2021), publication country (China vs. other), multicenter study (yes vs. no), sample size (≥200 vs. <200), post-NCRT features (whether post-NCRT MRI images were included), multi-MRI field strength (whether multiple field strengths were used), multi-MRI sequence (whether multiple sequences were used), ROI structure (2D vs. 3D), contour consistency (whether ROI delineation consistency was evaluated), feature extraction software (Pyradiomics vs. others), feature quantity after feature dimensionality reduction (≥10 vs. <10), multi-modeling algorithm (whether multiple methods were used for model construction), model validation (external validation vs. others), radiomics type (deep learning-based vs. machine learning-based), and RQS score (≥14 vs. <14).

Deeks’ asymmetry test was used to assess statistical significance, and potential publication bias was evaluated by plotting Deeks’ funnel plot. The trim-and-fill method was used to calculate the publication bias when a significant publication bias was found. Fagan plot was used to assess the clinical utility of MRI-based radiomics for predicting pCR in LARC patients after NCRT. All P-values under 0.05 were considered statistically significant.

## Results

### Literature search

**Figure 1:**
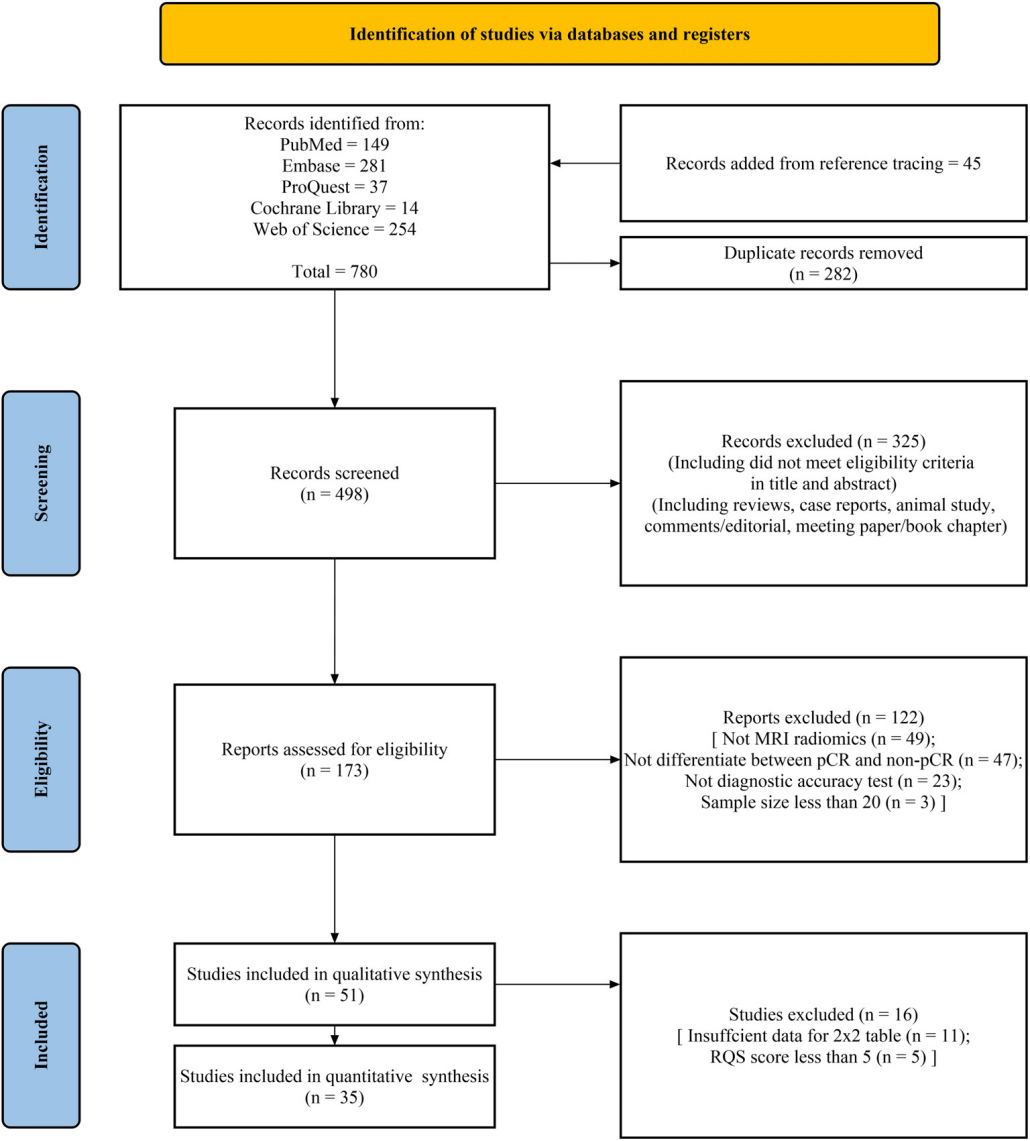
PRISMA flow chart of the study selection procedure for this systematic review and meta-analysis.

A total of 735 studies were retrieved through online search of PubMed, Embase, Proquest, Cochrane Library, and Web of Science databases, with an additional 45 articles identified through reference tracing. Detailed search results were shown in **Supplementary Table S1**. Following the removal of 282 duplicate studies, the titles and abstracts of the remaining articles were screened, and 325 articles were excluded. Subsequently, 122 articles were excluded for not meeting eligibility criteria. Eleven articles were excluded due to the inability to construct a 2x2 table, and five articles were excluded due to low RQS scores. Ultimately, 35 articles were included in this systematic review for analyses (13–47). The literature selection process is shown in **Figure 1**.

### Characteristics of included studies

**Table 1** showed the general characteristics of the studies included in the systematic review. The 35 studies, published between 2017 and 2024, included a total of 9,696 patients, with sample sizes ranging from 38 to 1,033. Among them, 2,102 cases were pCR patients, and 7,594 were non-pCR patients. The training sets included 5,822 cases, and the validation sets included 3,874 cases. The overall average age was 58.6 years, with a range from 50.5 to 70 years. Approximately 69% of studies (24/35) were published in 2021 or later. About 57% of studies were conducted in China (13, 15, 17, 19, 22–26, 28, 30, 33, 37, 40–45, 47) (20/35), five in the United States (18, 20, 32, 35, 38), four in Italy (16, 31, 36, 39), three in South Korea (27, 29, 34), and one each in Belgium, Brazil, and Turkey (14, 21, 46). Approximately 54% of studies were monocentric studies (19/35), 10 studies included data from two centers, four studies included data from three centers, and two studies included data from four centers. Most studies (approximately 83%) were retrospective, with three studies being prospective. Additionally, three studies conducted both retrospective model construction and validation, as well as prospective validation of the predictive performance of models. The magnetic field strength of scanners was 3.0T in 16 studies, accounting for about 46% (16/35), 1.5T in 5 studies, a combination of both in 12 studies, with one study using 1.5T and 1.0T and not reporting in one study. T2-weighted imaging (T2WI) and diffusion weighted imaging (DWI) were commonly used MRI sequences, accounting for 94% and 57%, respectively. Two studies used diffusion kurtosis imaging (DKI). The above imaging sequences were also often used simultaneously. Slice thicknesses of 3.0mm, 4.0mm, and 5.0mm were the most commonly used, accounting for 80%. Seventeen studies (about 49%) used pre-NCRT MRI images to predict pCR, four studies used images acquired after NCRT, and 13 studies used both pre- and post- NCRT images. Notably, one study used MRI images taken before, during, and after NCRT. Six studies utilized deep learning-based radiomics for analysis. The 2x2 table for the included studies were shown in **Supplementary Table S4**.

**Table 1:** The general characteristic of included studies.

First Author	Year	Country	Study Type and Centers	Sample Size (pCR/non-pCR)	Train/Validation Cohorts	Age	Chemoradiotherapy Regimen	Image Acquisition Period	Tesla	Sequence	Slice Thickness
Liu	2017	China	Retrospective (1 center)	222 (38/184)	152/70	55.9	50Gy/25 times; Cape	Before and After NCRT	3.0	T2WI/DWI	3.0mm,4.0mm
Horvat	2018	Brazil	Retrospective (1 center)	114 (21/93)	114/0	55.0	NR	After NCRT	3.0/1.5	T2WI/DWI	3.0mm,5.0mm
Cui	2018	China	Retrospective (1 center)	186 (31/155)	131/55	54.0	50Gy/25 times; Cape	Before NCRT	3.0	T2WI/CT1WI/DWI	3.0mm,5.0mm
Ferrari	2019	Italy	Prospective (1 center)	55 (16/39)	28/27	64.5	NR	Before, During and After NCRT	3.0	T2WI	4.0mm
Yi	2019	China	Retrospective (1 center)	134 (32/102)	93/41	51.0	46-50Gy/23-25 times; Cape	Before NCRT	3.0/1.5	T2WI	NR
Shaish	2020	USA	Retrospective (2 centers)	132 (20/112)	112/20	63.0	NR	Before NCRT	3.0/1.5	T2WI	3.00-8.00mm
Shao	2020	China	Retrospective (4 centers)	981 (240/741)	303/678	55.0	50Gy/25 times; Cape/CapeOX	Before NCRT	3.0/1.5	T2WI/DWI	3.0mm,4.0mm,5.0mm,6.0mm,8.0mm
Antunes	2020	USA	Retrospective (3 centers)	104 (23/81)	60/44	62.0	45-50.4Gy/25 times; Cape	Before NCRT	3.0/1.5	T2WI	3.0-8.00mm
Bulens	2020	Belgium	Prospective (2 centers)	125 (34/91)	70/55	64.0	45-50Gy/25 times; 5-FU/cape	Before and After NCRT	3.0	T2WI/DWI	3.0-5.0mm
Huang	2020	China	Retrospective (1 center)	270 (63/207)	236/34	62.0	45-50.4Gy/25 times; FOLFOX/5-FU	After NCRT	NR	NR	NR
Zhang	2020	China	Prospective (2 centers)	383 (74/309)	290/93	57.0	45Gy/22 times; Cape	Before and After NCRT	3.0	T1WI/T2WI/DKI	NR
Wan	2021	China	Retrospective (1 center)	165 (27/138)	116/49	54.0	45-50.4 Gy/25 times; CapeOX	Before and After NCRT	3.0	T2WI/DWI	3.0mm,4.0mm,5.0mm
Li	2021	China	Retrospective (2 centers)	80 (15/65)	80/0	56.5	50.4 Gy/25-28 times; Cape	Before NCRT	3.0	T2WI	3.0mm,4.0mm
Cheng	2021	China	Retrospective (1 center)	193 (31/162)	128/65	NR	45.0-50.4 Gy/25 times; mFOLFOX6, CapeOX	Before NCRT	3.0	T1WI/T2WI	3.0mm,4.0mm
Jang	2021	Republic of Korea	Retrospective (1 center)	466 (80/386)	353/113	60.0	50.4 Gy/25 times; 5-FU	After NCRT	3.0/1.5	T1WI/T2WI	3.0mm
Jin	2021	China	Retrospective and Prospective (2 centers)	624 (144/480)	323/301	54.0	NR	Before and After NCRT	3.0/1.5	T1WI/T2WI/DWI	3.0mm,5.0mm
Lee	2021	Republic of Korea	Retrospective (1 center)	912 (192/720)	592/320	59.0	NR	Before NCRT	3.0/1.5	T2WI/CT1WI/DWI	3.0mm
Pang	2021	China	Retrospective (2 centers)	187 (50/137)	107/80	56.0	45Gy/25 times; 5-FU	Before NCRT	1.5	T2WI	5.0mm
Boldrini	2022	Italy	Retrospective (3 centers)	59 (10/49)	0/59	60.0	45Gy/25 times; CapeOX	Before NCRT	1.5	T2WI	NR
Bordron	2022	USA	Retrospective (2 centers)	124 (14/110)	64/60	65.0	NR	Before NCRT	1.5/1.0	T2WI/DWI	3.0mm,3.5mm,4.0mm
Feng	2022	China	Retrospective and Prospective (4 centers)	1033 (244/789)	303/730	55.0	50Gy/25 times; 5-FU	Before NCRT	3.0/1.5	T2WI/CT1WI/DWI	2.0-6.0mm
Shin	2022	Republic of Korea	Retrospective (1 center)	898 (189/709)	592/306	59.0	NR	After NCRT	3.0/1.5	T2WI/DWI	3.0mm
Jayaprakasam	2022	USA	Retrospective (1 center)	236 (41/195)	236/0	54.0	50Gy/25-28 times; 5-FU/Cape	Before NCRT	3.0/1.5	T2WI	3.0mm
Nardone	2022	Italy	Retrospective (3 centers)	100 (21/79)	37/63	70.0	45Gy/25 times; Cape	Before and After NCRT	1.5	T2WI/DWI	NR
Zhang	2022	China	Retrospective (1 center)	38 (13/25)	26/12	61.0	50.6 Gy/22 times; PD-1 antibody and CapeOX	Before and After NCRT	3.0	T2WI/DWI	3.0mm,4.0mm
Horvat	2022	USA	Retrospective (2 centers)	164 (29/135)	114/50	59.0	NR	Before NCRT	3.0/1.5	T2WI/DWI	3.0mm,5.0mm
Chiloiro	2023	Italy	Retrospective (1 center)	203 (54/149)	134/69	65.0	45 Gy/25 times; CapeOX	Before and After NCRT	1.5	T2WI	NR
Huang	2023	China	Retrospective (2 centers)	563 (129/434)	362/201	57.0	45-46 Gy/25 times; 5-FU	Before and After NCRT	3.0	T2WI/DWI	2.0mm,3.0mm,4.0mm,5.0mm
Peng	2023	China	Retrospective (1 center)	83 (22/61)	83/0	64.0	450Gy/25 times; CapeOX	Before and After NCRT	3.0	T2WI/DWI/T1WI/CT1WI	3.0mm
Peng^a^	2023	China	Retrospective (1 center)	165 (26/139)	115/50	55.0	45-50.4 Gy/25 times; Cape	Before and After NCRT	3.0	T2WI/DWI/T1WI	3.0mm,4.0mm,5.0mm
Shi	2023	China	Retrospective and Prospective (3 centers)	224 (57/167)	147/77	58.5	45-50Gy/25 times; 5-FU	Before NCRT	3.0	T2WI/DWI/CT1WI	2.2mm,5.0mm,6.0mm
Wei	2023	China	Retrospective (2 centers)	151 (33/118)	100/51	55.5	45-50.4 Gy/25-28 times; CapeOX/FOLFOX/Cape/5-FU	Before and After NCRT	3.0	T2WI/DWI	3.0mm,4.0mm,5.0mm
Wen	2023	China	Retrospective (1 center)	126 (28/98)	84/42	50.5	45-50 Gy/25 times; CapeOX; mFOLFOX6/FOLFOX6	Before and After NCRT	3.0	T2WI	3.0mm
Yardimci	2023	Turkey	Retrospective (1 center)	76 (23/53)	53/23	58.0	45Gy/18 times; Cape	Before NCRT	1.5	T2WI	3.0mm
Ma	2024	China	Retrospective (1 center)	120 (38/82)	84/36	62.0	NR	Before NCRT	3.0	DKI/DWI	5.5mm

NR, Not reported; T1WI, T1 weighted imaging; T2WI, T2 weighting imaging; DWI, Diffusion weighted imaging; CT1WI, Contrast T1 weighted imaging; DKI, Diffusion kurtosis imaging; CapeOX, capecitabine plus oxaliplatin; Cape, capecitabine; 5-FU, 5-ﬂuorouracil; FOLFOX6, 5-ﬂuorouracil plus leucovorin plus oxaliplatin; mFOLFOX6, Modified 5-ﬂuorouracil plus leucovorin plus oxaliplatin; PD-1, Programmed cell death protein-1.

Detailed radiomics characteristics of the included studies were mentioned in **Table 2**. Eleven studies used ITK-SNAP software for radiomic feature extraction, and 7 used 3D Slicer. Approximately 77% of studies manual delineated the region of interest (ROI) (27/35), followed by automated (1/35) and semi-automated (2/35) delineation, with 3 studies not reporting and 2 not applicable. The ROI structure was 3D in 20 studies, 2D in 14 studies, and unspecified in 1 study. Most of studies (approximately 63%) conducted contour consistency assessments for ROI delineation. Pyradiomics was the most commonly used feature extraction software (14/35), followed by MATLAB (6/35). The vast majority of studies (29/35) extracted texture features, with first-order statistics (27/35), shape features (19/35), and wavelet features (11/35) also frequently extracted. In 24 studies, the number of features decreased by more than 90% after dimensionality reduction. Ten studies used the Z-score method for feature normalization, while 18 studies did not report the method. About 40% of the studies used least absolute shrinkage and selection operator (LASSO) for feature dimensionality reduction and selection (14/35). Similarly, logistic regression (LR) was the most commonly used algorithm for model construction (15/35), followed by support vector machine (SVM) (11/35), random forest (RF) (10/35), and neural networks (8/35). About 40% of studies performed external validation (14/35), followed by split sample (13/35) and cross-validation (11/35).

**Table 2:** Detailed radiomics characteristic of included studies.

First Author	ROI Software	ROI Segmentation	ROI Structure	Contour Consistency	Feature Extraction Software	Imaging Features	Feature Quantity (before and after dimensionality reduction)	Normalization	Feature Dimensionality Reduction and Selection	Modeling Algorithm	Model Validation	Optimal Model	AUC
Liu	ITK-SNAP	manual	3D	yes	MATLAB	First order/Texture/Wavelet	2252/30	Z-score	T test/LASSO	SVM	Split sample	SVM	0.94
Horvat	ITK-SNAP	manual	3D	no	MATLAB	Texture	34/14	NR	Gini index	RF	FivefoldCV	RF	0.93
Cui	ITK-SNAP	manual	2D	yes	Analysis Kit	First Order/Texture	1188/12	Z-score	LR/LASSO/PCC	LR	Split sample	LR	0.94
Ferrari	3D Slicer	manual	2D	no	Python	First Order/Texture/Area	855/8	GreyLevel Intensity Normalization	T test	RF	Split sample	RF	0.86
Yi	MaZda	manual	2D	yes	MaZda	First Order/Texture/Wavelet	340/10	NR	Wilcoxon rank-sum test/Chi-square test/LASSO	RF/SVM	Split sample	Combined	0.93
Shaish	3D Slicer	manual	3D	yes	Pyradiomics	First Order/Texture/Wavelet/Shape/Square/Square root	3190/40	NR	PCC/RFE	LR	FivefoldCV	LR	0.80
Shao	ITK-SNAP	manual	2D	yes	Pyradiomics	First Order/Texture/Wavelet/Shape/LOG	770/7	Z-score	Spearman test	XGBoost	External validation	XGBoost	0.95
Antunes	3D Slicer	manual	2D	NR	MATLAB	Texture	764/4	Mean	mRMR/LASSO	RF	External validation	RF	0.71
Bulens	NR	manual	2D	yes	NR	NR	8524/NR	NR	PCA/LASSO	LR-LASSO	External validation	LR-LASSO	0.86
Huang	NR	NR	NR	NR	NR	NR	NR	NR	NR	ANN	Split sample	ANN	0.84
Zhang	ITK-SNAP	manual	2D	NR	NR	NR	NR	NR	NR	CNN	External validation	CNN	0.99
Wan	Radcloud	manual	3D	yes	Radcloud	First Order/Texture/Shape/High order	1049/10	NR	LASSO	LR	TenfoldCV	LR	0.91
Li	Radcloud	manual	3D	yes	Radcloud	First Order/Texture/Shape/High order	1049/11	NR	LASSO/PCA	LR/DT/KNN/RF	External validation	KNN	0.95
Cheng	ITK-SNAP	manual	3D	yes	Pyradiomics	First Order/Shape/Texture/Wavelet/LOG/LG	1967/7	NR	Mann-Whitney U test/LASSO	LR	Split sample	LR	0.91
Jang	NA	NA	3D	NR	MATLAB	NA	NR	NR	NR	CNN	Split sample	CNN	0.76
Jin	NA	NA	3D	yes	NR	NA	NA	NA	NA	3D RP-Net	External validation	3D RP-Net	0.95
Lee	3D Slicer	semi-automatic	3D	NR	Pyradiomics	First Order/Shape/Texture	3740/9	Z-score	PCA	LR/XGBoost/LightGBM/RF/MLP/Ensemble	Split sample	RF	0.84
Pang	U-Net	automatic	2D	no	Pyradiomics	First Order/Texture/Shape/Wavelet	2370/10	NR	Harrell’s concordance index	SVM	External validation	SVM	0.83
Boldrini	NR	NR	3D	NR	Moddicom	First Order/Shape/LOG	1200/2	NR	LOG	LR	External validation	Combined	0.75
Bordron	3D Slicer	manual	3D	yes	Miras	First Order/Shape/Texture	822/3	COMBAT	PCC	ANN	External validation	Combined	0.81
Feng	ITK-SNAP	manual	2D	yes	Pyradiomics	First Order/Texture/Shape/High order	3740/9	Z-score	Mann-Whitney U test/LASSO/RFE	SVM	External validation	SVM	0.79
Shin	3D Slicer	semi-automatic	2D	yes	Pyradiomics	First Order/Texture/Shape/High order	1132/19	Z-score	Spearman correlation coefficient/LASSO	LR	TenfoldCV	LR	0.82
Jayaprakasam	Gold LX	manual	3D	yes	MATLAB	First Order/Texture	101/11	NR	Mann-Whitney nonparametric test	SVM	FivefoldCV	SVM	0.89
Nardone	LifeX Software	manual	3D	yes	LifeX Software	First Order/Shape/Texture	13/3	NR	Mann-Whitney nonparametric test	LR	Split sample	LR	0.87
Zhang	NR	manual	2D	yes	MATLAB	First Order/Shape/Texture	164/2	NR	T test/LASSO	Linear prediction model	ThreefoldCV	Linear prediction model	0.86
Horvat	ITK-SNAP	manual	3D	yes	NR	Texture/Haralick texture/Gabor edge	NR/91	NR	NR	RF	External validation	RF	0.83
Chiloiro	Eclipse	manual	3D	no	Moddicom	First Order/Shape/Texture	546/2	NR	PCC	LR	Split sample	LR	0.80
Huang	Darwin	manual	2D	yes	Pyradiomics	First Order/Shape/Texture	NR/21	NR	T test/LASSO/RFE	SVM	TenfoldCV and External validation	SVM	0.86
Peng	ITK-SNAP	NR	3D	yes	Pyradiomics	First Order/Shape/Texture/Wavelet	3720/8	Z-score	AONVA/mRMR/GBDT	SVM/RF/KNN/LR	Split sample	KNN	0.91
Peng^a^	Radcloud	manual	2D	yes	Pyradiomics	First Order/Shape/Texture/Wavelet	5636/3	Z-score	mRMR/LASSO	LR	TenfoldCV	LR	0.89
Shi	MIM Maestro	manual	3D	NR	Pyradiomics	First Order/Texture	428/14	COMBAT	PCA	SVM	FivefoldCV and External validation	SVM	0.87
Wei	ITK-SNAP	manual	3D	NR	Pyradiomics	First Order/Texture/Wavelet/LOG	4492/11	Intensity of image was scaled to 0-255	ICC/LASSO	RF/SVM/LR/KNN/XGBoost	TenfoldCV and External validation	RF	0.83
Wen	MaZda	manual	3D	yes	NR	First Order/Texture	250/12	Z-score	PCC/AONVA/Relief	GP/AdaBoost	Split sample	GP	0.91
Yardimci	3D Slicer	manual	3D	NR	Pyradiomics	First Order/Texture/Shape/Wavelet/LOG	1046/8	Z-score	AONVA/RFE/Kruskal-Wallis/Relief	LR/SVM/RF/ANN	TenfoldCV	RF	0.75
Ma	ITK-SNAP	manual	2D	yes	Pyradiomics	First Order/Shape/Texture/Wavelet/LOG	1046/NR	MinMax	SelectKBest	SVM	Split sample	SVM	0.83

NR, Not reported; NA, Not applicable; LOG, Laplacian of Gaussian; LG, Logarithm; mRMR, minimum redundancy maximum relevancy; PCC, Pearson’s correlation coefficient; PCA, Principal component analysis; LASSO, Least absolute shrinkage and selection operator; ICC, Interclass correlation coefficient; RFE, Recursive feature elimination; ANOVA, Analysis of variance; GBDT, Gradient boosting decision tree; ANN, Artificial neural network; CNN, Convolutional neural network; SVM, Support vector machine; RF, Random forest; LR, Logistic regression; XGBoost, eXtreme Gradient Boosting; DT, Decision tree; KNN, K-nearest neighbor; GP, Gaussian process; Combined, Joint prediction model of radiomics and clinical indicators; CV, Cross validation.

### Quality assessment

#### QUADAS-2

**Figure 2:**
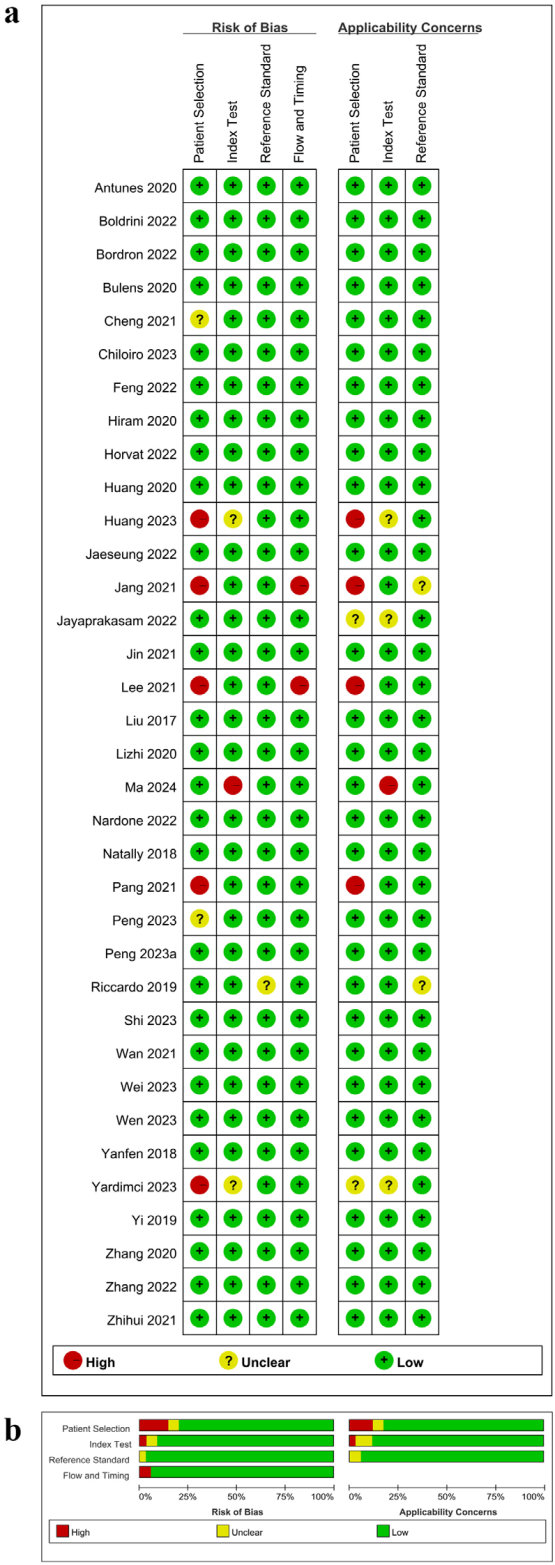
Risk of bias and applicability concerns according to Quality Assessment of Diagnostic Accuracy Studies-2 tool. **(a)** Per study assessment; **(b)** Per domain summary.

According to QUADAS-2, the risk of bias and applicability concerns for the included studies were shown in **Figure 2**. In the patient selection domain, the overall risk of bias was relatively low (<25%). Only a few studies showed a high risk of bias due to not specifying the timeframe for case inclusion or difficulty in determining whether the study was case-controlled (**Figure 2a**). Additionally, the applicability concerns in this domain were generally low (**Figure 2b**). Two studies did not explicitly specify the severity of rectal cancer in patients, and four studies lacked relevant research background or relatively intact demographic characteristics. Similarly, the overall risk of bias in the index test domain was less than 25%, although 1 study owned a high risk of bias due to not using a predefined threshold. Three studies did not report whether the index test was conducted with blinding, leading to some applicability concerns in this domain. Since the treatment efficacy of NCRT for all study subjects included was confirmed through postoperative histopathological biopsy, the risk of bias in the reference standard domain was relatively low. Furthermore, postoperative histopathological biopsy is considered as the gold standard for determining pCR (1), resulting in low applicability concerns in this domain. Two studies did not specify the time interval between MRI examinations and the reference standard. However, the overall risk of bias in the flow and timing domain was low, as all cases in the included studies were subject to radiomics analysis. Detailed quality assessment results were shown in **Supplementary Table S5**. Taken together, the quality of the articles included was acceptable according to the QUADAS-2 assessment.

#### RQS

**Figure 3:**
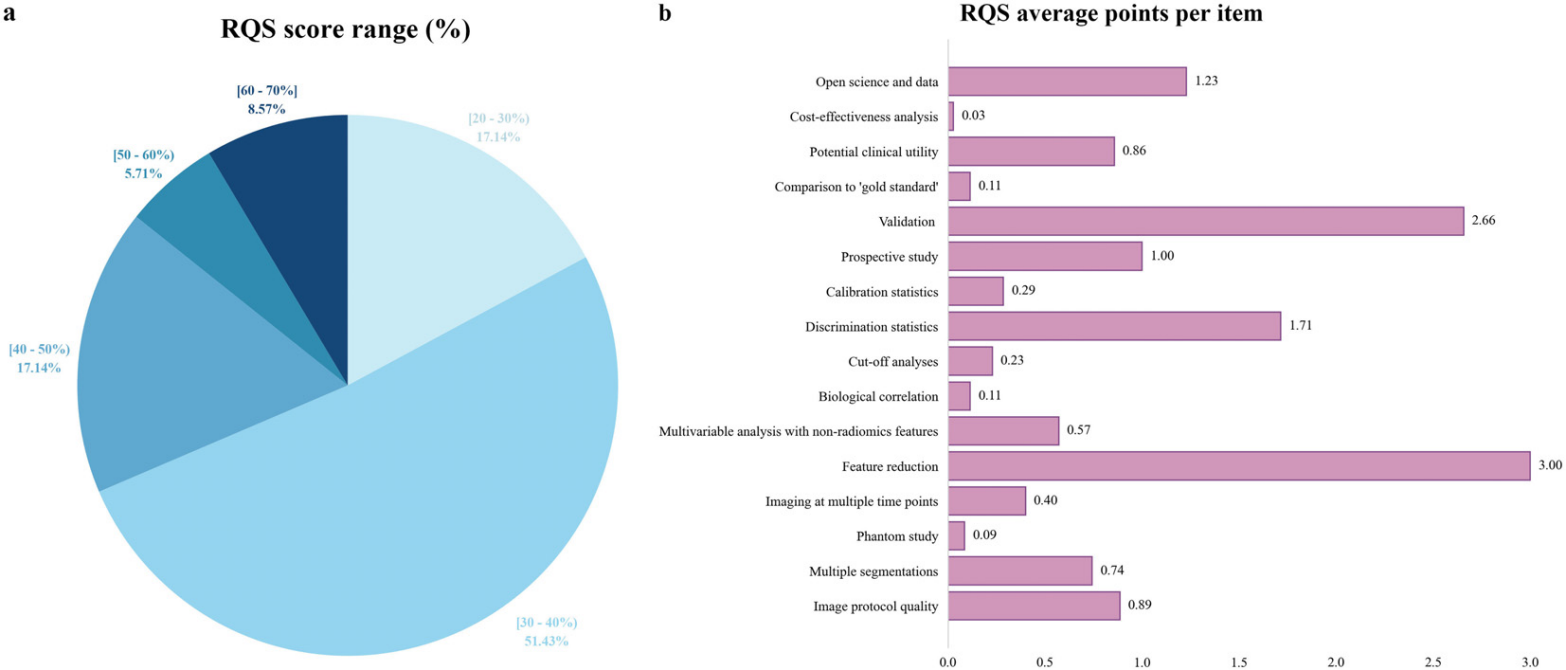
Methodological quality assessment based on the RQS tool. **(a)** Proportion of studies per different RQS range percentages. **(b)** Average points per each RQS item. RQS, radiomics quality score.

The average RQS score of 35 included studies was 13.91, approximately 38.64% of the total score. The median score was 13, with a range from 9 (25%) to 24 (67%). Over half of the studies (about 51%) scored between 30% and 40% (**Figure 3**). All included studies conducted “Feature reduction or adjustment for multiple testing”, “Discrimination statistics”, “Validation” and “Open science and data” items. Additionally, 75% and over 86% of studies conducted “Multiple segmentations” and “Well-documented image protocols” items respectively. Approximately 58% of studies performed multivariable analysis, incorporating non-radiomics features, which is expected to provide a more holistic model. About 44% of studies reported potential clinical utility and provided clinical decision curves. Only one study conducted a cost-effectiveness analysis for the clinical application of the model. Only five studies registered prospective cohort studies in trial databases, providing the highest level of evidence supporting the clinical validity and usefulness of the radiomics biomarker. Only three studies conducted phantom studies, which help detect inter-scanner differences and vendor-dependent features. Detailed RQS scores for all included studies were provided in **Supplementary Table S6**.

### Meta-analysis

#### Heterogeneity analysis

The threshold effect across studies was examined by calculating the Spearman correlation coefficient between the logit of sensitivity and logit of 1-specificity. The results showed a Spearman correlation coefficient of 0.119 (P = 0.498), with the scatter points corresponding to the included studies distributing in a non- “shoulder arm” pattern on the sROC curve, indicating no significant threshold effect. The I^2^ statistic indicated significant heterogeneity in sensitivity (I^2^ = 78.5%, P < 0.001) and specificity (I^2^ = 92.1%, P < 0.001) across the study cohorts.

#### Diagnostic test accuracy analysis

**Figure 4:**
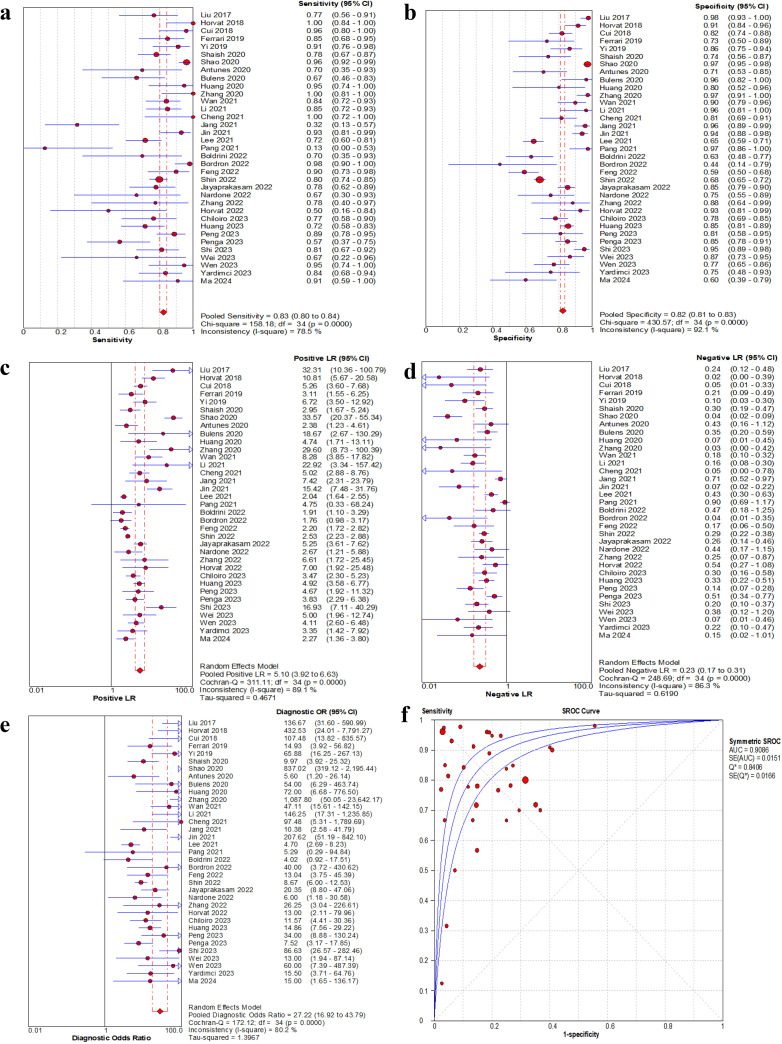
Forest plot of sensitivity, specificity, positive LR, negative LR and DOR, and sROC curve of MRI-based radiomics for prediction of pathological complete response. **(a)** Sensitivity for MRI. **(b)** Specificity for MRI. **(c)** Positive LR for MRI. **(d)** Negative LR for MRI. **(e)** DOR for MRI. **(f)** The sROC plane for heterogeneity test of threshold effect of each independent study. Corresponding indices, 95% CI and the pooled indices are represented by red circles, horizontal lines and red diamonds, respectively. The sROC (middle line) with 95% CI (the other two lines). LR, likelihood ratio; DOR, diagnostic odds ratio; sROC, summary receiver operator characteristic; AUC, area under the curve; Q^*^, Q index value; SE, standard error; CI, confidence interval.

A total of 35 studies were included in this meta-analysis, and only the validation or test cohorts with superior predictive performance were evaluated. The pooled sensitivity, specificity, positive likelihood ratio, negative likelihood ratio and DOR were 0.83 (95% CI: 0.80-0.84), 0.82 (95% CI: 0.81-0.83), 5.10 (95% CI: 3.92-6.63), 0.23 (95% CI: 0.17-0.31), and 27.22 (95% CI: 16.92-43.79), respectively. The AUC was 0.91. The forest plots and sROC curve for the pooled effect sizes were shown in **Figure 4**.

#### Heterogeneity exploration and meta-regression

As shown in **Table 3**, 15 covariates were used to explore potential sources of heterogeneity. Meta-regression and joint model analysis indicated the following factors as contributors to significant heterogeneity in the meta-analysis: publication year (≥ 2021 vs. < 2021) (P = 0.02), publication country (China vs. others) (P = 0.01), multi-magnetic field strength (multi-Telsa vs. mono-Telsa) (P = 0.02), multi-MRI sequence (multi-sequence vs. mono-sequence) (P = 0.01), ROI structure (3D vs. 2D) (P = 0.02), contour consistency (evaluated vs. unevaluated) (P < 0.001), feature extraction software (Pyradiomics vs. others) (P < 0.001), and feature quantity after feature dimensionality reduction (≥ 10 vs. <10) (P < 0.001).

**Table 3:** Investigation of heterogeneity using meta-regression and subgroup analysis.

Covariates		N	Sensitivity	*P_1_*	Specificity	*P_2_*	Joint Model Analysis		
							I^2^	LRT chi^2^	*P*
Year	≥2021	24	0.80 [0.73 - 0.87]	0.00	0.83 [0.78 - 0.89]	0.00	74 [43 - 100]	7.70	0.02
	<2021	11	0.90 [0.84 - 0.96]		0.90 [0.85 - 0.95]				
Country	China	15	0.78 [0.69 - 0.87]	0.00	0.80 [0.72 - 0.88]	0.00	78 [53 - 100]	9.26	0.01
	Others	20	0.87 [0.82 - 0.93]		0.89 [0.85 - 0.93]				
Multicenter	Yes	15	0.83 [0.74 - 0.91]	0.01	0.89 [0.84 - 0.94]	0.01	0 [0 - 100]	1.78	0.41
	No	20	0.84 [0.78 - 0.91]		0.83 [0.77 - 0.89]				
Sample size	≥200	13	0.84 [0.76 - 0.92]	0.02	0.89 [0.83 - 0.94]	0.01	0 [0 - 100]	1.65	0.44
	<200	22	0.83 [0.77 - 0.90]		0.84 [0.78 - 0.89]				
Post-NCRT features	Yes	15	0.85 [0.77 - 0.92]	0.02	0.86 [0.80 - 0.92]	0.00	0 [0 - 100]	0.12	0.94
	No	20	0.83 [0.76 - 0.90]		0.86 [0.80 - 0.91]				
Multi-MRI Telsa	Yes	14	0.85 [0.77 - 0.92]	0.04	0.84 [0.77 - 0.91]	0.00	76 [46 - 100]	8.20	0.02
	No	20	0.82 [0.74 - 0.89]		0.87 [0.82 - 0.92]				
Multi-MRI sequence	Yes	23	0.85 [0.79 - 0.91]	0.13	0.88 [0.83 - 0.92]	0.03	80 [57 - 100]	10.02	0.01
	No	11	0.80 [0.69 - 0.90]		0.82 [0.73 - 0.91]				
ROI structure	3D	20	0.83 [0.76 - 0.90]	0.02	0.86 [0.81 - 0.91]	0.00	74 [42 - 100]	7.67	0.02
	2D	14	0.83 [0.75 - 0.92]		0.86 [0.79 - 0.92]				
Contour consistency	Yes	22	0.86 [0.80 - 0.92]	0.88	0.86 [0.80 - 0.91]	0.07	98 [97 - 99]	109.40	0.00
	No	4	0.77 [0.58 - 0.97]		0.88 [0.77 - 0.99]				
Feature extraction software	Pyradiomics	14	0.81 [0.72 - 0.90]	0.01	0.83 [0.76 - 0.90]	0.00	96 [93 - 99]	52.52	0.00
	Others	16	0.85 [0.77 - 0.92]		0.85 [0.78 - 0.91]				
Feature quantity	≥10	15	0.81 [0.74 - 0.89]	0.00	0.89 [0.84 - 0.93]	0.08	97 [95 - 99]	74.31	0.00
	<10	14	0.85 [0.78 - 0.92]		0.78 [0.70 - 0.86]				
Multi-modeling algorithm	Yes	7	0.86 [0.75 - 0.96]	0.13	0.83 [0.72 - 0.94]	0.01	0 [0 - 100]	0.58	0.75
	No	28	0.83 [0.77 - 0.89]		0.87 [0.82 - 0.91]				
Model validation	External	14	0.83 [0.74 - 0.92]	0.01	0.90 [0.85 - 0.95]	0.02	23 [0 - 100]	2.59	0.27
	Others	21	0.84 [0.77 - 0.91]		0.83 [0.77 - 0.89]				
Deep learning	Yes	6	0.91 [0.83 - 0.99]	0.59	0.90 [0.81 - 0.98]	0.14	38 [0 - 100]	3.22	0.20
	No	29	0.82 [0.76 - 0.88]		0.85 [0.80 - 0.90]				
RQS	≥14	16	0.88 [0.82 - 0.93]	0.11	0.89 [0.84 - 0.94]	0.01	65 [22 - 100]	5.74	0.06
	<14	19	0.79 [0.71 - 0.87]		0.83 [0.77 - 0.89]				

#### Subgroup analysis

**Figure 5:**
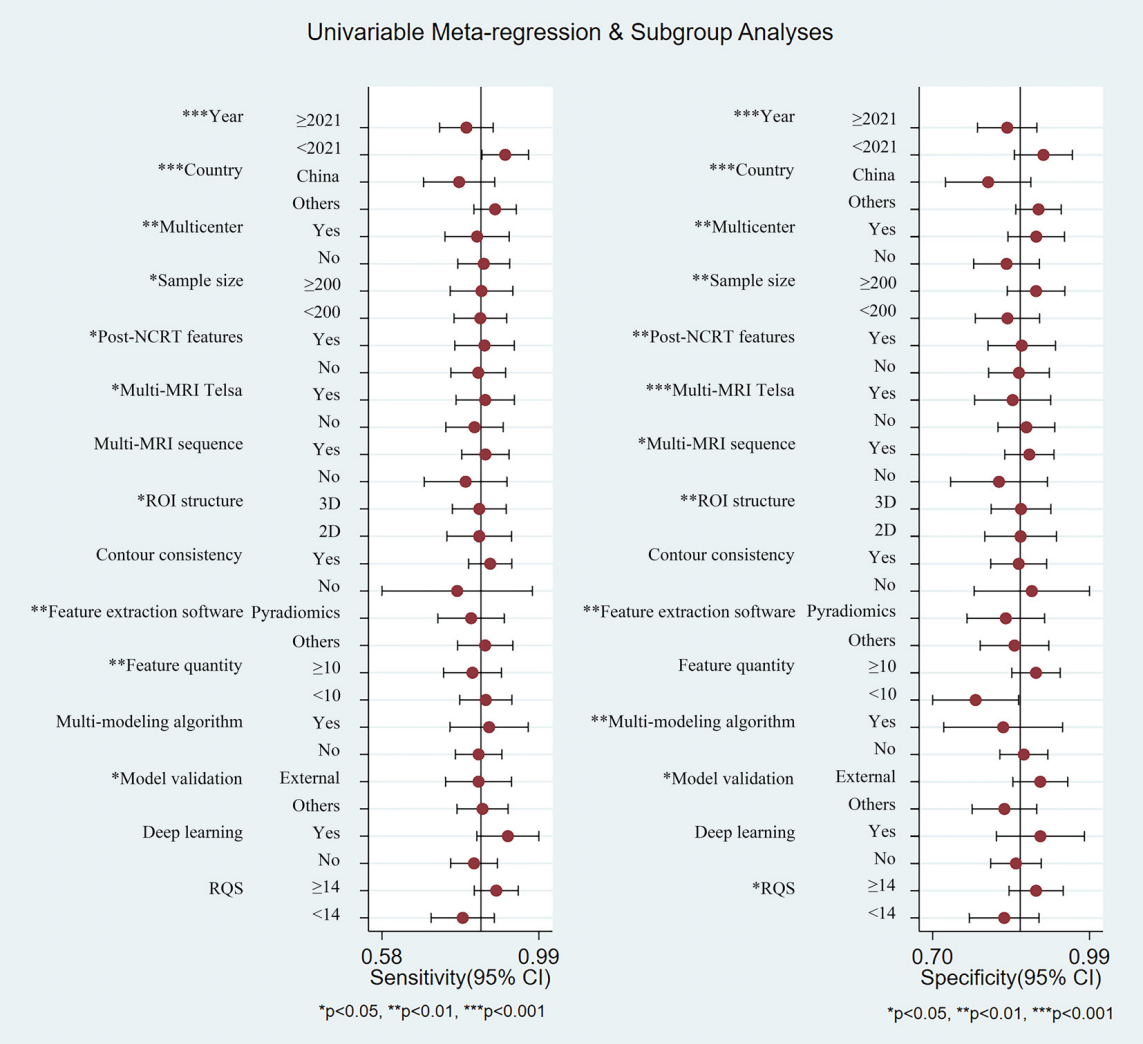
Summary plot of sensitivity and specificity of each subgroup using univariable meta-regression and subgroup analyses.

According to subgroup analysis results (**Figure 5**), studies from China showed lower pooled sensitivity (78% vs. 87%, P < 0.001) and specificity (80% vs. 89%, P < 0.001) compared to studies from other countries. Studies using multicenter cohorts for model development and validation owned higher specificity (89% vs. 83%, P = 0.01). Studies with a sample size of 200 or more demonstrated higher sensitivity (84% vs. 83%, P = 0.02) and specificity (89% vs. 84%, P = 0.01). Studies incorporating multiple MRI imaging sequences showed higher sensitivity (85% vs. 80%, P = 0.13, not significant) and specificity (88% vs. 82%, P = 0.03) compared to those using only one sequence. Studies that performed consistency evaluations for lesion contouring showed higher sensitivity (86% vs. 77%, P = 0.88, not significant), which helped analyze the robustness of radiomic features to segmentation variability. Studies with using Pyradiomics software for feature extraction showed lower sensitivity (81% vs. 85%, P = 0.01) and specificity (83% vs. 85%, P < 0.001). Studies that validated the predictive performance of the model on external validation cohorts manifested lower sensitivity (83% vs. 84%, P = 0.01) and higher specificity (90% vs. 83%, P = 0.02). Deep learning-based radiomics studies showed higher sensitivity (91% vs. 82%, P = 0.59, not significant) and specificity (90% vs. 85%, P = 0.14, not significant) compared to conventional radiomics, although these differences were not statistically significant. From the overall score, studies with higher RQS scores demonstrated higher sensitivity (88% vs. 79%, P = 0.11, not significant) and specificity (89% vs. 83%, P = 0.01).

#### Publication bias

**Figure 6:**
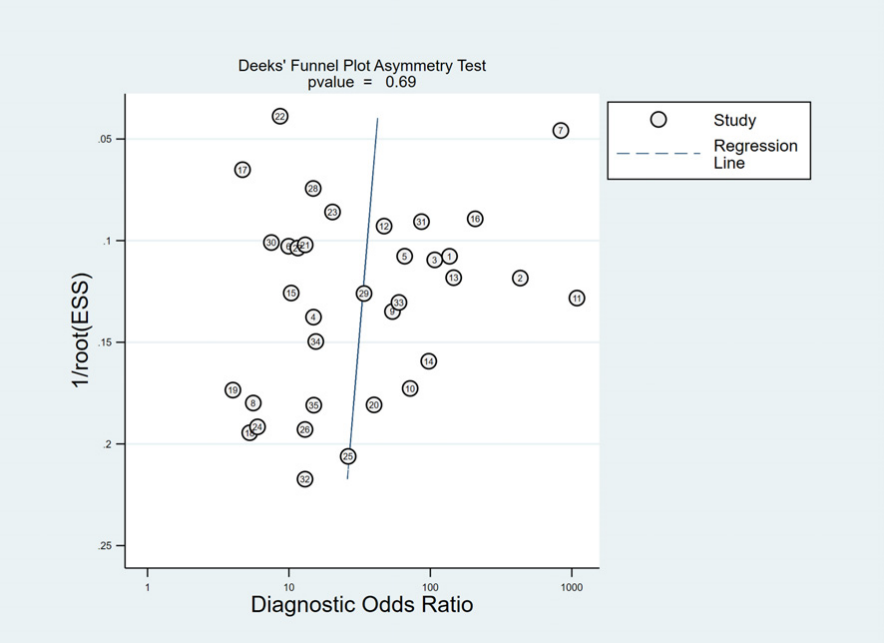
The Deeks’ funnel plot asymmetry test for publication bias in the literature evaluation. Each study is shown as a circle, and the regression line is shown.

Deeks’ test was used to explore potential publication bias in the included studies. The funnel plot (**Figure 6**) showed a generally symmetrical distribution around the regression line, suggesting no significant publication bias (P = 0.69).

#### Clinical utility

**Figure 7:**
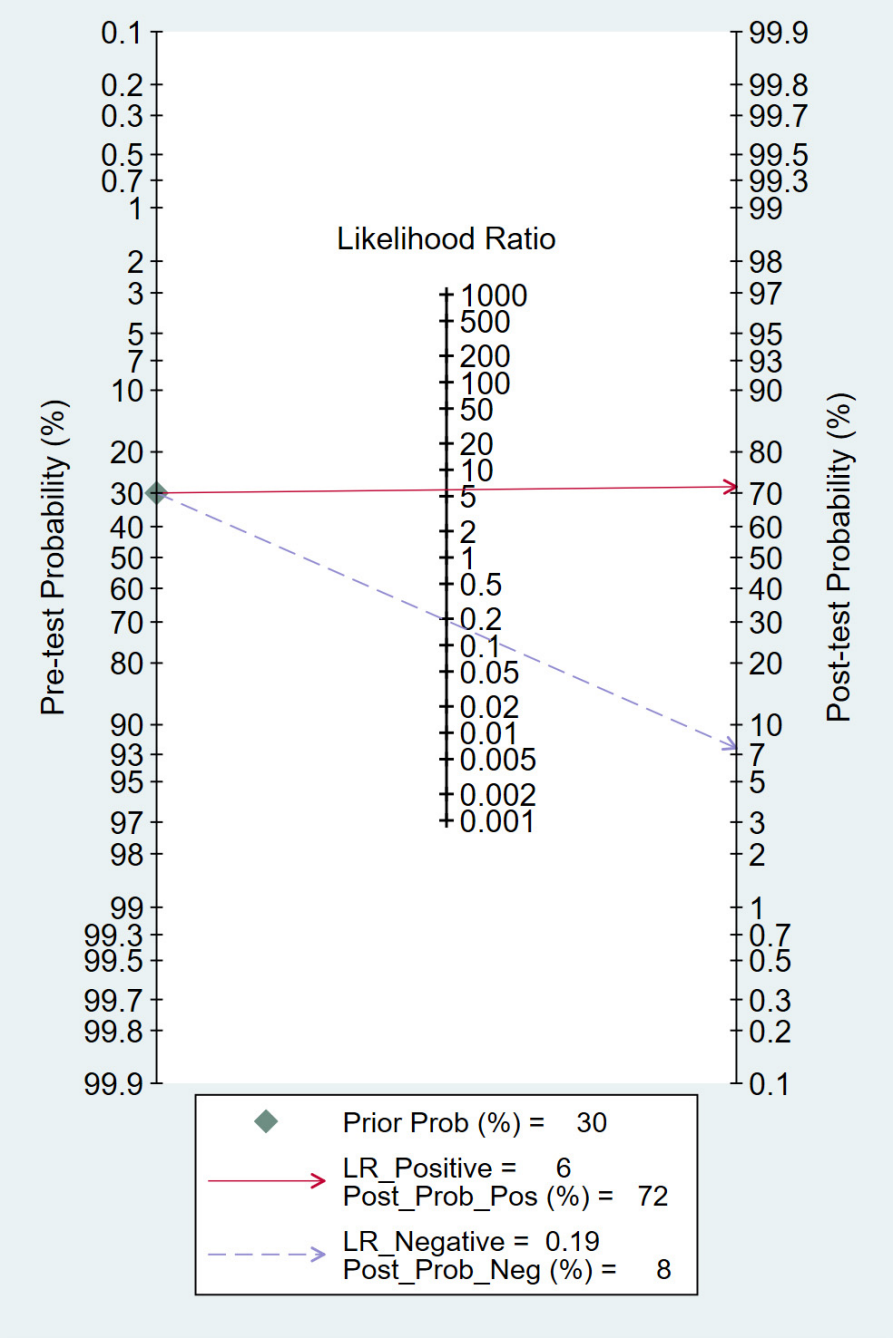
Fagan plots for assessing clinical utility.

According to the Fagan plot of the study cohort (**Figure 7**), the pre-test probability of predicting pCR in LARC patients was 30%. The positive likelihood ratio for MRI radiomics in predicting pCR was 6, and the negative likelihood ratio was 0.19, which increased the post-test probability of a positive result to 72% and decreased the post-test probability of a negative result to 8%.

## Discussion

Accurate assessment of NCRT efficacy in LARC patients after receiving NCRT can provide critical information for subsequent treatment decisions. Studies have shown that if accurate prediction of pCR in LARC patients receiving NCRT could be made preoperatively, a “watch and wait” strategy may be adopted. This approach could help avoid surgical complications and permanent stoma formation, ultimately helping to improve quality of life and achieve precision medicine (48). Currently, the predictive value of conventional imaging methods for pCR is limited (6, 7), whereas the efficacy of pCR prediction has been significantly improved by MRI-based radiomics in recent years. By extracting numerous features from MRI images, identifying correlations between image features and predictive outcomes, and using machine learning methods to build predictive models, MRI-based radiomics offers a new approach for predicting pCR (49).

This study conducted a meta-analysis of 35 relevant studies, showing that MRI-based radiomics owned a high AUC (0.91) for predicting pCR in LARC patients following NCRT, with pooled sensitivity and specificity of 0.83 (95% CI: 0.80-0.84) and 0.82 (95% CI: 0.81-0.83), respectively. These findings are consistent with previous reviews and systematic reviews (50, 51). The Fagan plot demonstrated that MRI-based radiomics can raise the post-test probability of a positive result to 72% and reduce the post-test probability of a negative result to 8%. Compared to previous studies using conventional imaging methods for pCR prediction, MRI-based radiomics showed significantly enhanced predictive performance, indicating that MRI-based radiomics provided a new approach for predicting pCR and may offer valuable guidance for treatment planning in LARC patients (52). Compared with the previous study by Jia et al., the predictive performance of MRI radiomics for pCR is largely consistent. However, this study demonstrated superiority from 4 aspects: (1) an increased number of included studies (35 vs. 21); (2) higher quality of included studies (RQS 13.91 vs. 10.95); (3) exploration of potential sources of heterogeneity using meta-regression; and (4) additional subgroup analysis (9).

We assessed the quality of included studies using the QUADAS-2 and RQS tools. Since all study subjects were confirmed by postoperative histopathological biopsy, the risk of bias and overall applicability concerns in the reference standard domain were low. Some studies did not clearly specify whether cases were enrolled consecutively, performed ROI delineation without implementing blinding, or did not report the interval between MRI examination and pathological biopsy, resulting in certain risks of bias and overall applicability concerns in domains other than the reference standard. Phantom study was designed to detect potential feature differences across scanners and vendors. Many studies used MRI imaging data from different vendors (e.g., Siemens, Philips) and multiple medical centers, making phantom study a suitable approach to measure these uncertainties and identify vendor-dependent features. Cost-effectiveness analysis was a health economics approach that advocated for cost-quality-adjusted life-year comparison, with or without the use of radiomics, to more accurately evaluate the economic potential of such studies. Future study reports should consider incorporating the above details of quality assessment.

We emphasized the role of various feature extraction software, including Pyradiomics, in contributing to the heterogeneity of results. This variability stemmed from the diversity of algorithmic approaches and parameter settings across software. While not inherently detrimental but rather highlighted the subtle impact of the choice of feature extraction software on research outcomes. Given this variability, we underscored the importance of understanding and transparently reporting the software used, as it significantly impacted on the overall radiomics analysis. Likewise, contour consistency is another critical source of heterogeneity that cannot be ignored. Evaluating the consistency of ROI delineation helped to ensure high concordance in the outlining of lesion contours among different observers or by the same observer at different time points. This was crucial for radiomics studies, as feature extraction relied on precise lesion regions. Large variations in contour could lead to fluctuations in extracted features, thereby impacting stability and predictive performance of model. Different observers may interpret lesion boundaries differently, especially when the lesion morphology is complex, or boundaries are indistinct. These discrepancies can affect the conclusions of individual studies and potentially introduce greater bias in multicenter studies, thereby undermining the quality of evidence.

Subgroup analysis results indicated that studies published in 2021 or later showed slightly lower predictive performance compared to those published before 2021. The reasons may be analyzed as a result of the lack of robust validation, resulting in potential model overfitting. Studies with a sample size greater than 200, using multicenter cohorts, incorporating post-NCRT MRI features, or employing multiple MRI sequences, showed higher predictive performance. This could be that larger sample sizes allow for more comprehensive training and validation of the model, and richer imaging information provides a greater chance of extracting features that can accurately distinguish pCR from non-pCR during the feature extraction phase, thus better reflecting the efficacy of NCRT and enhancing model predictive performance. Deep learning-based radiomics studies showed higher sensitivity (91% vs. 82%, P = 0.59, not significant) and specificity (90% vs. 85%, P = 0.14, not significant) than those of machine learning-based, though the differences were not statistically significant. In terms of feature extraction, traditional machine learning methods typically rely on manually designed and selected features. This requires experts to predetermine useful features based on domain knowledge, such as edges or textures in image processing. This process is time-consuming, depends on expert experience, and may miss some potentially important features. Deep learning, particularly convolutional neural networks, can automatically extract multi-level features from raw data. Through multiple layers of nonlinear transformation, deep learning models can detect complex and abstract patterns in data without the need of manually designed features. This automated feature extraction not only improves efficiency but also reveals deep features that traditional methods may overlook. The primary characteristic of deep learning methods is the emphasis on feature learning, namely autonomously learning data representations (53). This is the key distinction between deep learning and more “traditional” machine learning methodologies. However, this meta-analysis included only six deep learning studies, and more research is needed to confirm this conclusion. Additionally, studies with RQS scores ≥14 demonstrated higher predictive performance compared to those with RQS <14, suggesting that studies with a more standardized radiomics workflow have fewer confounding factors, which reduces bias while enhances the predictive performance and robustness of the model.

While current systematic review has focused specifically on locally advanced rectal cancer (LARC), it is noteworthy that colorectal cancer (CRC) as a broader entity remains the third most prevalent malignancy worldwide, with colon cancer accounting for approximately 70% of all CRC cases (54). The integration of the Internet of Things (IoT) into surgical practice has revolutionized CRC management by enabling real-time data acquisition, remote monitoring, and enhanced intraoperative precision. IoT-driven devices, such as smart surgical instruments and wearable biosensors, facilitate continuous postoperative surveillance of physiological parameters (e.g. bowel motility, inflammatory markers) to detect complications early. Furthermore, IoT platforms enhance multidisciplinary collaboration by synchronizing imaging, pathology, and clinical data, thereby optimizing preoperative planning and personalized therapeutic strategies (55).

Deep learning algorithms have emerged as transformative tools in CRC diagnosis, particularly in histopathology image analysis. Recent studies demonstrate that convolutional neural networks can classify colorectal adenocarcinoma with accuracy comparable to expert pathologists, reducing interobserver variability and diagnostic delays (56). For example, Deep learning models trained on whole-slide images (WSIs) excel in detecting subtle morphological features, such as tumor budding and lymph vascular invasion, which are critical for staging and prognosis. Bousis et al. highlighted that Deep learning-based systems achieve >90% sensitivity in differentiating benign polyps from malignant lesions, minimizing unnecessary biopsies (57). Similarly, Chlorogiennis et al. emphasized the utility in predicting microsatellite instability status directly from H&E-stained slides, potentially bypassing costly molecular testing (58).

Postoperative complications, such as infection, anastomotic leakage, bowel obstruction, and postoperative bleeding, remain significant challenges in colorectal surgery, impacting morbidity and long-term survival. Emerging biomarkers, including butyrylcholinesterase (BChE), show promise in predicting these adverse outcomes. BChE, an enzyme involved in detoxification and inflammation modulation, correlates with systemic stress responses. Recent evidence suggests that preoperative BChE levels inversely associate with postoperative ileus and sepsis risk, potentially reflecting impaired cholinergic anti-inflammatory pathways (59). Patients with low BChE activity (<1,900 IU/L) exhibit a higher risk of anastomotic dehiscence, likely due to dysregulated tissue repair and prolonged inflammation (60). While these findings underscore the prognostic utility of BChE, further validation in prospective cohorts is needed to establish standardized cutoff values and evaluate interactions with comorbidities (e.g. hepatic dysfunction). Combining BChE with clinical risk scores may refine perioperative decision-making, enabling targeted interventions for high-risk patients. Overall, despite these advances, clinical implementation faces hurdles, including model generalizability across diverse populations and regulatory standardization. Future integration with radiomics may yield multimodal predictive frameworks, further enhancing CRC management.

This systematic review and meta-analysis has several limitations that are necessary to mention: (1) Most of the included studies were retrospectively designed (approximately 83%). Prospective studies are generally regarded as superior to retrospective studies due to standardized imaging protocols, timely and relevant radiomic feature extraction, standardized and blinded data collection, and optimized study design. These factors can all enhance the quality and relevance of the study results. (2) Only a small number of studies used deep learning methods for model establishment. Deep learning and convolutional neural network offer a more automated and efficient approach to feature extraction, allowing for extracting high-level features from images. This makes them particularly suitable for radiomics studies, where the complexity and volume of medical imaging data can be high. (3) There was substantial heterogeneity among studies due to non-threshold effects (for sensitivity, I^2^ = 78.5%, P < 0.001). Although meta-regression was used to explore numerous potential sources of heterogeneity, it was not possible to identify all sources. (4) We only evaluated pCR studies and did not include studies on tumor regression grading and T downstaging. It is known that pathological evaluation of TRG and T downstaging is more subjective than pCR evaluation. (5) Only the predictive performance of MRI-based radiomics models and their combination with clinical factors were evaluated. In actual clinical practice, other imaging modalities (e.g., PET-CT, ultrasound), as well as pathological and biochemical indicators, are commonly used to assess NCRT efficacy, relying on a multidisciplinary comprehensive judgment.

## Conclusion

In summary, MRI-based radiomics demonstrates a high efficacy in predicting whether LARC patients achieve pCR following NCRT, providing valuable guidance for treatment planning and potentially enhancing patients’ quality of life. However, there is still a lack of large-sample, prospective, multicenter external validation studies, and the external applicability of MRI-based radiomics models requires further investigation. With the advancement of big data and resource sharing, alongside the innovation and enhancement of computational and artificial intelligence methodologies, the predictive performance of MRI-based radiomics for pCR stands to be significantly augmented, holding promise for the realization of precision medicine tailored to patients with LARC.

## Data Availability

The original contributions presented in the study are included in the article/**Supplementary Material**. Further inquiries can be directed to the corresponding author.
