# Supplementary material for: MRI-based radiomics for predicting pathological complete response after neoadjuvant chemoradiotherapy in locally advanced rectal cancer: a systematic review and meta-analysis
Source: Front Oncol. 2025 Mar 10;15:1550838. doi: 10.3389/fonc.2025.1550838 (PMC11930822; doi:10.3389/fonc.2025.1550838)
Supplement: Supplementary file 1 [file Table1.docx]

# Supplementary Material

Supplementary material for “MRI-based radiomics for predicting pathological complete response after neoadjuvant chemoradiotherapy in locally advanced rectal cancer: a systematic review and meta-analysis”, including Table S1-S6.

### Table S1 Literature searching strategies in PubMed, Embase, Cochrane Library and Web of science

| **Search** | **PubMed Query - July 1 2024** | **Items found** |
| --- | --- | --- |
| #9 | #1 AND #5 AND #6 AND #7 AND #8 | 149 |
| #8 | "pathological complete response"[Title/Abstract] OR "complete pathological response"[Title/Abstract] OR "complete pathologic response"[Title/Abstract] OR "pathologic complete response"[Title/Abstract] OR "tumor regression grade"[Title/Abstract] OR "treatment response"[Title/Abstract] OR "response to therapy"[Title/Abstract] OR "response to treatment"[Title/Abstract] OR "tumor response"[Title/Abstract] OR "pathological response"[Title/Abstract] OR "complete response"[Title/Abstract] OR "neoadjuvant chemoradiation therapy"[Title/Abstract] OR "rectal cancer response"[Title/Abstract] OR "rectal cancer treatment response"[Title/Abstract] | 151,006 |
| #7 | "Rectal Neoplasms"[MeSH Terms] OR "Colorectal Neoplasms"[MeSH Terms] OR "locally advanced rectal cancer"[Title/Abstract] OR "rectal cancer"[Title/Abstract] OR "locally advanced rectal carcinoma"[Title/Abstract] OR "rectal carcinoma"[Title/Abstract] OR "Rectal Neoplasms"[Title/Abstract] OR "Colorectal Neoplasms"[Title/Abstract] OR "rectal tumor"[Title/Abstract] | 258,031 |
| #6 | "Neoadjuvant Therapy"[MeSH Terms] OR "Neoadjuvant Therapy"[Title/Abstract] OR "neoadjuvant chemoradiotherapy"[Title/Abstract] OR "neoadjuvant chemotherapy"[Title/Abstract] OR "neoadjuvant radiotherapy"[Title/Abstract] | 50,166 |
| #5 | #2 OR #3 OR #4 | 817,987 |
| #4 | "machine learning"[Title/Abstract] OR "deep learning"[Title/Abstract] OR "supervised machine learning"[Title/Abstract] OR "supervised learning"[Title/Abstract] OR "unsupervised machine learning"[Title/Abstract] OR "unsupervised learning"[Title/Abstract] OR "support vector machine"[Title/Abstract] OR "decision tree"[Title/Abstract] OR "logistic"[Title/Abstract] OR "neural network"[Title/Abstract] OR "convolutional neural network"[Title/Abstract] OR "CNN"[Title/Abstract] | 753,677 |
| #3 | "Machine Learning"[MeSH Terms] OR "Deep Learning"[MeSH Terms] OR "Supervised Machine Learning"[MeSH Terms] OR "Support Vector Machine"[MeSH Terms] OR "Unsupervised Machine Learning"[MeSH Terms] | 77,557 |
| #2 | "Radiomics"[MeSH Terms] OR "Radiomics"[Title/Abstract] OR "radiomic analysis"[Title/Abstract] OR "radiogenomics"[Title/Abstract] OR "radiopathomics"[Title/Abstract] OR "texture analysis"[Title/Abstract] OR "texture"[Title/Abstract] OR "LASSO"[Title/Abstract] | 73,409 |
| #1 | "Magnetic Resonance Imaging"[MeSH Terms] OR "MRI"[Title/Abstract] | 693,953 |

| **Search** | **Embase Query - July 1 2024** | **Items found** |
| --- | --- | --- |
| #9 | #1 AND #5 AND #6 AND #7 AND #8 | 281 |
| #8 | ("pathological complete response" OR "complete pathological response" OR "complete pathologic response" OR "pathologic complete response" OR "tumor regression grade" OR "treatment response" OR "response to therapy" OR "response to treatment" OR "tumor response" OR "pathological response" OR "complete response" OR "neoadjuvant chemoradiation therapy" OR "rectal cancer response" OR "rectal cancer treatment response"):ab,ti | 264,324 |
| #7 | ("Rectal Neoplasms" OR "Colorectal Neoplasms")/exp OR ("locally advanced rectal cancer" OR "rectal cancer" OR "locally advanced rectal carcinoma" OR "rectal carcinoma" OR "Rectal Neoplasms" OR "Colorectal Neoplasms" OR "rectal tumor"):ab,ti | 507,528 |
| #6 | "Neoadjuvant Therapy"/exp OR ("Neoadjuvant Therapy" OR "neoadjuvant chemoradiotherapy" OR "neoadjuvant chemotherapy" OR "neoadjuvant radiotherapy"):ab,ti | 92,475 |
| #5 | #2 OR #3 OR #4 | 1,331,155 |
| #4 | ("machine learning" OR "deep learning" OR "supervised machine learning" OR "supervised learning" OR "unsupervised machine learning" OR "unsupervised learning" OR "support vector machine" OR "decision tree" OR "logistic" OR "neural network" OR "convolutional neural network" OR "CNN"):ab,ti | 988,208 |
| #3 | ("Machine Learning" OR "Deep Learning" OR "Supervised Machine Learning" OR "Support Vector Machine" OR "Unsupervised Machine Learning")/exp | 519,125 |
| #2 | "Radiomics"/exp OR ("Radiomics" OR "radiomic analysis" OR "radiogenomics" OR "radiopathomics" OR "texture analysis" OR "texture" OR "LASSO"):ab,ti | 83,244 |
| #1 | "magnetic resonance imaging"/exp OR mri:ab,ti | 1,385,132 |

| **Search** | **ProQuest Query - July 1 2024** | **Items found** |
| --- | --- | --- |
| #9 | #1 AND #5 AND #6 AND #7 AND #8 | 37 |
| #8 | MESH("pathological complete response") OR MESH("complete pathological response") OR MESH("complete pathologic response") OR MESH("pathologic complete response") OR MESH("tumor regression grade") OR MESH("treatment response") OR MESH("response to therapy") OR MESH("response to treatment") OR MESH("tumor response") OR MESH("pathological response") OR MESH("complete response") OR MESH("neoadjuvant chemoradiation therapy") OR MESH("rectal cancer response") OR MESH("rectal cancer treatment response") | 58,662 |
| #7 | MESH("Rectal Neoplasms") OR MESH("Colorectal Neoplasms") OR SUMMARY("locally advanced rectal cancer") OR SUMMARY("rectal cancer") OR SUMMARY("locally advanced rectal carcinoma") OR SUMMARY("rectal carcinoma") OR SUMMARY("Rectal Neoplasms") OR SUMMARY("Colorectal Neoplasms") OR SUMMARY("rectal tumor") | 21,697 |
| #6 | MESH("Neoadjuvant Therapy") OR SUMMARY("Neoadjuvant Therapy") OR SUMMARY("neoadjuvant chemoradiotherapy") OR SUMMARY("neoadjuvant chemotherapy") OR SUMMARY("neoadjuvant radiotherapy") | 13,293 |
| #5 | #2 OR #3 OR #4 | 1,189,144 |
| #4 | SUMMARY("machine learning") OR SUMMARY("deep learning") OR SUMMARY("supervised machine learning") OR SUMMARY("supervised learning") OR SUMMARY("unsupervised machine learning") OR SUMMARY("unsupervised learning") OR SUMMARY("support vector machine") OR SUMMARY("decision tree") OR SUMMARY("logistic") OR SUMMARY("neural network") OR SUMMARY("convolutional neural network") OR SUMMARY("CNN") | 1,000,698 |
| #3 | MESH("Machine Learning") OR MESH("Deep Learning") OR MESH("Supervised Machine Learning") OR MESH("Support Vector Machine") OR MESH("Unsupervised Machine Learning") | 14 |
| #2 | MESH("Radiomics") OR SUMMARY("Radiomics") OR SUMMARY("radiomic analysis") OR SUMMARY("radiogenomics") OR SUMMARY("radiopathomics") OR SUMMARY("texture analysis") OR SUMMARY("texture") OR SUMMARY("LASSO") | 207,334 |
| #1 | MESH("Magnetic Resonance Imaging") OR SUMMARY(MRI) | 243,653 |

| **Search** | **Cochrane Library Query - July 1 2024** | **Items found** |
| --- | --- | --- |
| #21 | #3 AND #12 AND #15 AND #19 AND #20 | 14 |
| #20 | ("pathological complete response" OR "complete pathological response" OR "complete pathologic response" OR "pathologic complete response" OR "tumor regression grade" OR "treatment response" OR "response to therapy" OR "response to treatment" OR "tumor response" OR "pathological response" OR "complete response" OR "neoadjuvant chemoradiation therapy" OR "rectal cancer response" OR "rectal cancer treatment response"):ti,ab,kw | 46,586 |
| #19 | #16 OR #17 OR #18 | 15,428 |
| #18 | ("locally advanced rectal cancer" OR "rectal cancer" OR "locally advanced rectal carcinoma" OR "rectal carcinoma" OR "Rectal Neoplasms" OR "Colorectal Neoplasms" OR "rectal tumor"):ti,ab,kw | 13,439 |
| #17 | MeSH descriptor: [Colorectal Neoplasms] explode all trees | 12,914 |
| #16 | MeSH descriptor: [Rectal Neoplasms] explode all trees | 2,922 |
| #15 | #13 OR #14 | 10,055 |
| #14 | ("Neoadjuvant Therapy" OR "neoadjuvant chemoradiotherapy" OR "neoadjuvant chemotherapy" OR "neoadjuvant radiotherapy"):ti,ab,kw | 10,055 |
| #13 | MeSH descriptor: [Neoadjuvant Therapy] explode all trees | 2,700 |
| #12 | #4 OR #5 OR #6 OR #7 OR #8 OR #9 OR #10 OR #11 | 41,541 |
| #11 | ("machine learning" OR "deep learning" OR "supervised machine learning" OR "supervised learning" OR "unsupervised machine learning" OR "unsupervised learning" OR "support vector machine" OR "decision tree" OR "logistic" OR "neural network" OR "convolutional neural network" OR "CNN"):ti,ab,kw | 38,880 |
| #10 | MeSH descriptor: [Unsupervised Machine Learning] explode all trees | 5 |
| #9 | MeSH descriptor: [Support Vector Machine] explode all trees | 64 |
| #8 | MeSH descriptor: [Supervised Machine Learning] explode all trees | 83 |
| #7 | MeSH descriptor: [Deep Learning] explode all trees | 336 |
| #6 | MeSH descriptor: [Machine Learning] explode all trees | 1,023 |
| #5 | ("Radiomics" OR "radiomic analysis" OR "radiogenomics" OR "radiopathomics" OR "texture analysis" OR "texture" OR "LASSO"):ti,ab,kw | 3,408 |
| #4 | MeSH descriptor: [Radiomics] explode all trees | 53 |
| #3 | #1 OR #2 | 42,371 |
| #2 | (MRI):ti,ab,kw | 36,432 |
| #1 | MeSH descriptor: [Magnetic Resonance Imaging] explode all trees | 13,155 |

| **Search** | **Web of Science Query - July 1 2024** | **Items found** |
| --- | --- | --- |
| #9 | #1 AND #5 AND #6 AND #7 AND #8 and Preprint Citation Index (Exclude – Database) | 254 |
| #8 | TS=("pathological complete response" OR "complete pathological response" OR "complete pathologic response" OR "pathologic complete response" OR "tumor regression grade" OR "treatment response" OR "response to therapy" OR "response to treatment" OR "tumor response" OR "pathological response" OR "complete response" OR "neoadjuvant chemoradiation therapy" OR "rectal cancer response" OR "rectal cancer treatment response") and Preprint Citation Index (Exclude – Database) | 245,287 |
| #7 | TS=("locally advanced rectal cancer" OR "rectal cancer" OR "locally advanced rectal carcinoma" OR "rectal carcinoma" OR "Rectal Neoplasms" OR "Colorectal Neoplasms" OR "rectal tumor") and Preprint Citation Index (Exclude – Database) | 264,645 |
| #6 | TS=("Neoadjuvant Therapy" OR "neoadjuvant chemoradiotherapy" OR "neoadjuvant chemotherapy" OR "neoadjuvant radiotherapy") and Preprint Citation Index (Exclude – Database) | 84,716 |
| #5 | #2 OR #3 OR #4 and Preprint Citation Index (Exclude – Database) | 3,999,151 |
| #4 | TS=("machine learning" OR "deep learning" OR "supervised machine learning" OR "supervised learning" OR "unsupervised machine learning" OR "unsupervised learning" OR "support vector machine" OR "decision tree" OR "logistic" OR "neural network" OR "convolutional neural network" OR "CNN") and Preprint Citation Index (Exclude – Database) | 3,462,201 |
| #3 | TS=("Machine Learning" OR "Deep Learning" OR "Supervised Machine Learning" OR "Support Vector Machine" OR "Unsupervised Machine Learning") and Preprint Citation Index (Exclude – Database) | 1,924,569 |
| #2 | TS=("Radiomics" OR "radiomic analysis" OR "radiogenomics" OR "radiopathomics" OR "texture analysis" OR "texture" OR "LASSO") and Preprint Citation Index (Exclude – Database) | 599,421 |
| #1 | TS=("Magnetic Resonance Imaging" OR "MRI") and Preprint Citation Index (Exclude – Database) | 1,222,675 |

### Table S2 Description of the Quality Assessment of Diagnostic Accuracy Studies (QUADAS-2) tool

| **DOMAIN** | **PATIENT SELECTION** | **INDEX TEST** | **REFERENCE STANDARD** | **FLOW AND TIMING** |
| --- | --- | --- | --- | --- |
| **Description** | **Describe methods of patient selection: Describe included patients (prior testing, presentation, intended use of index test and setting):** | **Describe the index test and how it was conducted and interpreted:** | **Describe the reference standard and how it was conducted and interpreted:** | **Describe any patients who did not receive the index test(s) and/or reference standard or who were excluded from the 2x2 table (refer to flow diagram): Describe the time interval and any interventions between index test(s) and reference standard:** |
| **Signaling questions**  **(yes/no/unclear)** | **Was a consecutive or random sample of patients enrolled?** | **Were the index test results interpreted without knowledge of the results of the reference standard?** | **Is the reference standard likely to correctly classify the target condition?** | **Was there an appropriate interval between index test(s) and reference standard?** |
|  | **Was a case-control design avoided?** | **If a threshold was used, was it pre-specified?** | **Were the reference standard results interpreted without knowledge of the results of the index test?** | **Did all patients receive a reference standard?** |
|  | **Did the study avoid inappropriate exclusions?** |  |  | **Did all patients receive the same reference standard?** |
|  |  |  |  | **Were all patients included in the analysis?** |
| **Risk of bias:**  **High/low/unclear** | **Could the selection of patients have introduced bias?** | **Could the conduct or interpretation of the index test have introduced bias?** | **Could the reference standard, its conduct, or its interpretation have introduced bias?** | **Could the patient flow have introduced bias?** |
| **Concerns regarding applicability:**  **High/low/unclear** | **Are there concerns that the included patients do not match the review question?** | **Are there concerns that the index test, its conduct, or interpretation differ from the review question?** | **Are there concerns that the target condition as defined by the reference standard does not match the review question?** |  |

### Table S3 Description of the radiomics quality score (RQS) tool

|  | **Criteria** | **Points** |
| --- | --- | --- |
| **1** | **Image protocol quality** - well-documented image protocols (for example, contrast, slice thickness, energy, etc.) and/or usage of public image protocols allow reproducibility/replicability | + 1 (if protocols are well-documented)  + 1 (if public protocol is used) |
| **2** | **Multiple segmentations** - possible actions are: segmentation by different physicians/algorithms/software, perturbing segmentations by (random) noise, segmentation at different breathing cycles. Analyse feature robustness to segmentation variabilities | + 1 |
| **3** | **Phantom study on all scanners** - detect inter-scanner differences and vendor-dependent features. Analyse feature robustness to these sources of variability | + 1 |
| **4** | **Imaging at multiple time points** - collect images of individuals at additional time points. Analyse feature robustness to temporal variabilities (for example, organ movement, organ expansion/ shrinkage) | + 1 |
| **5** | **Feature reduction** **or adjustment** for multiple testing - decreases the risk of overfitting. Overfitting is inevitable if the number of features exceeds the number of samples. Consider feature robustness when selecting features | - 3 (if neither measure is implemented)  + 3 (if either measure is implemented) |
| **6** | **Multivariable analysis with non radiomics features** (for example, EGFR mutation) - is expected to provide a more holistic model. Permits correlating/inferencing between radiomics and non radiomics features | + 1 |
| **7** | Detect and discuss **biological correlates** - demonstration of phenotypic differences (possibly associated with underlying gene–protein expression patterns) deepens understanding of radiomics and biology | + 1 |
| **8** | **Cut-off analyses** - determine risk groups by either the median, a previously published cut-off or report a continuous risk variable. Reduces the risk of reporting overly optimistic results | + 1 |
| **9** | **Discrimination statistics** - report discrimination statistics (for example, C-statistic, ROC curve, AUC) and their statistical significance (for example, p-values, confidence intervals). One can also apply resampling method (for example, bootstrapping, cross-validation) | + 1 (if a discrimination statistic and its statistical significance are reported)  + 1 (if a resampling method technique is also applied) |
| **10** | **Calibration statistics** - report calibration statistics (for example, Calibration-in-the-large/slope, calibration plots) and their statistical significance (for example, *P*-values, confidence intervals). One can also apply resampling method (for example, bootstrapping, cross-validation) | + 1 (if a calibration statistic and its statistical significance are reported)  + 1 (if a resampling method technique is also applied) |
| **11** | **Prospective study registered** in a trial database - provides the highest level of evidence supporting the clinical validity and usefulness of the radiomics biomarker | + 7 (for prospective validation of a radiomics signature in an appropriate trial) |
| **12** | **Validation** - the validation is performed without retraining and without adaptation of the cut-off value, provides crucial information with regard to credible clinical performance | - 5 (if validation is missing)  + 2 (if validation is based on a dataset from the same institute)  + 3 (if validation is based on a dataset from another institute)  + 4 (if validation is based on two datasets from two distinct institutes)  + 4 (if the study validates a previously published signature)  + 5 (if validation is based on three or more datasets from distinct institutes)  *Datasets should be of comparable size and should have at least 10 events per model feature |
| **13** | **Comparison to 'gold standard'** - assess the extent to which the model agrees with/is superior to the current ‘gold standard’ method (for example, TNM-staging for survival prediction). This comparison shows the added value of radiomics | + 2 |
| **14** | **Potential clinical utility** - report on the current and potential application of the model in a clinical setting (for example, decision curve analysis). | + 2 |
| **15** | **Cost-effectiveness analysis** - report on the cost-effectiveness of the clinical application (for example, QALYs generated) | + 1 |
| **16** | **Open science and data** - make code and data publicly available. Open science facilitates knowledge transfer and reproducibility of the study | + 1 (if scans are open source)  + 1 (if region of interest segmentations are open source)  + 1 (if code is open source)  + 1 (if radiomics features are calculated on a set of representative ROIs and the calculated features and representative ROIs are open source) |
|  | Total points (36=100%) |  |

### Table S4 The 2x2 table of included studies

| **First Author** | **True positive** | **False negative** | **False positive** | **True negative** |
| --- | --- | --- | --- | --- |
| Liu | 20 | 6 | 3 | 123 |
| Horvat | 21 | 0 | 8 | 85 |
| Cui | 24 | 1 | 23 | 103 |
| Ferrari | 28 | 5 | 6 | 16 |
| Yi | 31 | 3 | 8 | 51 |
| Shaish | 61 | 17 | 9 | 25 |
| Shao | 148 | 6 | 15 | 509 |
| Antunes | 7 | 3 | 10 | 24 |
| Bulens | 18 | 9 | 1 | 27 |
| Huang | 18 | 1 | 3 | 12 |
| Zhang | 18 | 0 | 2 | 73 |
| Wan | 48 | 9 | 6 | 53 |
| Li | 45 | 8 | 1 | 26 |
| Cheng | 11 | 0 | 10 | 44 |
| Jang | 6 | 13 | 4 | 90 |
| Jin | 40 | 3 | 7 | 109 |
| Lee | 56 | 22 | 85 | 157 |
| Pang | 1 | 7 | 1 | 37 |
| Boldrini | 7 | 3 | 18 | 31 |
| Bordron | 50 | 1 | 5 | 4 |
| Feng | 27 | 3 | 49 | 71 |
| Shin | 176 | 44 | 214 | 464 |
| Jayaprakasam | 32 | 9 | 29 | 166 |
| Nardone | 6 | 3 | 7 | 21 |
| Zhang | 7 | 2 | 2 | 15 |
| Horvat | 4 | 4 | 3 | 39 |
| Chiloiro | 23 | 7 | 23 | 81 |
| Huang | 38 | 15 | 45 | 264 |
| Peng | 56 | 7 | 4 | 17 |
| Peng^a^ | 17 | 13 | 20 | 115 |
| Shi | 35 | 8 | 5 | 99 |
| Wei | 4 | 2 | 6 | 39 |
| Wen | 18 | 1 | 15 | 50 |
| Yardimci | 31 | 6 | 4 | 12 |
| Ma | 10 | 1 | 10 | 15 |

### Table S5 Risk of bias and application concerns assessment of each study by the QUADAS-2 tool

| Study ID | Risk of Bias | | | | Applicability Concerns | | |
| --- | --- | --- | --- | --- | --- | --- | --- |
|  | Patient selection | Index test | Reference standard | Flow and timing | Patient selection | Index test | Reference standard |
| Antunes 2020 | Low | Low | Low | Low | Low | Low | Low |
| Boldrini 2022 | Low | Low | Low | Low | Low | Low | Low |
| Bordron 2022 | Low | Low | Low | Low | Low | Low | Low |
| Bulens 2020 | Low | Low | Low | Low | Low | Low | Low |
| Cheng 2021 | Unclear | Low | Low | Low | Low | Low | Low |
| Chiloiro 2023 | Low | Low | Low | Low | Low | Low | Low |
| Feng 2022 | Low | Low | Low | Low | Low | Low | Low |
| Hiram 2020 | Low | Low | Low | Low | Low | Low | Low |
| Horvat 2022 | Low | Low | Low | Low | Low | Low | Low |
| Huang 2020 | Low | Low | Low | Low | Low | Low | Low |
| Huang 2023 | High | Unclear | Low | Low | High | Unclear | Low |
| Jaeseung 2022 | Low | Low | Low | Low | Low | Low | Low |
| Jang 2021 | High | Low | Low | High | High | Low | Unclear |
| Jayaprakasam 2022 | Low | Low | Low | Low | Unclear | Unclear | Low |
| Jin 2021 | Low | Low | Low | Low | Low | Low | Low |
| Lee 2021 | High | Low | Low | High | High | Low | Low |
| Liu 2017 | Low | Low | Low | Low | Low | Low | Low |
| Lizhi 2020 | Low | Low | Low | Low | Low | Low | Low |
| Ma 2024 | Low | High | Low | Low | Low | High | Low |
| Nardone 2022 | Low | Low | Low | Low | Low | Low | Low |
| Natally 2018 | Low | Low | Low | Low | Low | Low | Low |
| Pang 2021 | High | Low | Low | Low | High | Low | Low |
| Peng 2023 | Unclear | Low | Low | Low | Low | Low | Low |
| Peng^a^ 2023 | Low | Low | Low | Low | Low | Low | Low |
| Ricardo 2019 | Low | Low | Unclear | Low | Low | Low | Unclear |
| Shi 2023 | Low | Low | Low | Low | Low | Low | Low |
| Wan 2021 | Low | Low | Low | Low | Low | Low | Low |
| Wei 2023 | Low | Low | Low | Low | Low | Low | Low |
| Wen 2023 | Low | Low | Low | Low | Low | Low | Low |
| Yanfen 2018 | Low | Low | Low | Low | Low | Low | Low |
| Yardimci 2023 | High | Unclear | Low | Low | Unclear | Unclear | Low |
| Yi 2019 | Low | Low | Low | Low | Low | Low | Low |
| Zhang 2020 | Low | Low | Low | Low | Low | Low | Low |
| Zhang 2022 | Low | Low | Low | Low | Low | Low | Low |
| Zhihui 2021 | Low | Low | Low | Low | Low | Low | Low |

### Table S6 Methodology quality assessment of each study by the RQS tool

| Study ID | Image protocol quality | Multiple segmentations | Phantom study on all scanners | Imaging at multiple time points | Feature reduction | Multivariable analysis | Biological correlation | Cut-off analyses | Discrimination statistics | Calibration statistics | Prospective study | Validation | Comparison to 'gold standard' | Potential clinical utility | Cost-effectiveness analysis | Open science and data | Total points |
| --- | --- | --- | --- | --- | --- | --- | --- | --- | --- | --- | --- | --- | --- | --- | --- | --- | --- |
| Antunes 2020 | 1 | 1 | 1 | 0 | 3 | 0 | 0 | 0 | 2 | 0 | 0 | 4 | 0 | 0 | 0 | 1 | 13  (36.11%) |
| Boldrini 2022 | 0 | 0 | 0 | 0 | 3 | 1 | 0 | 0 | 1 | 0 | 0 | 3 | 0 | 0 | 0 | 1 | 9  (25%) |
| Bordron 2022 | 1 | 1 | 0 | 0 | 3 | 1 | 0 | 1 | 2 | 0 | 0 | 3 | 0 | 2 | 0 | 1 | 15  (41.67%) |
| Bulens 2020 | 1 | 1 | 0 | 1 | 3 | 1 | 0 | 0 | 2 | 0 | 7 | 3 | 0 | 0 | 0 | 1 | 20  (55.56%) |
| Cheng 2021 | 1 | 0 | 0 | 0 | 3 | 1 | 0 | 0 | 2 | 1 | 0 | 2 | 0 | 2 | 0 | 1 | 13  (36.11%) |
| Chiloiro 2023 | 0 | 1 | 0 | 1 | 3 | 1 | 1 | 0 | 2 | 0 | 0 | 2 | 0 | 0 | 0 | 1 | 12  (33.33%) |
| Feng 2022 | 1 | 1 | 0 | 0 | 3 | 1 | 0 | 0 | 2 | 0 | 7 | 5 | 0 | 2 | 0 | 2 | 24  (66.67%) |
| Hiram 2020 | 1 | 1 | 0 | 0 | 3 | 1 | 0 | 0 | 2 | 0 | 0 | 3 | 0 | 0 | 0 | 1 | 12  (33.33%) |
| Horvat 2022 | 1 | 1 | 1 | 0 | 3 | 0 | 0 | 0 | 2 | 0 | 0 | 4 | 0 | 0 | 0 | 2 | 14  (38.89%) |
| Huang 2020 | 0 | 0 | 0 | 0 | 3 | 1 | 0 | 1 | 2 | 0 | 0 | 2 | 0 | 0 | 0 | 1 | 10  (27.78%) |
| Huang 2023 | 1 | 1 | 0 | 1 | 3 | 0 | 0 | 0 | 2 | 1 | 0 | 3 | 0 | 2 | 0 | 1 | 15  (41.67%) |
| Jaeseung 2022 | 1 | 1 | 1 | 0 | 3 | 0 | 0 | 1 | 1 | 0 | 0 | 2 | 0 | 2 | 0 | 2 | 14  (38.89%) |
| Jang 2021 | 1 | 0 | 0 | 0 | 3 | 0 | 0 | 0 | 1 | 0 | 0 | 2 | 0 | 0 | 1 | 1 | 9  (25%) |
| Jayaprakasam 2022 | 1 | 1 | 0 | 0 | 3 | 0 | 0 | 0 | 1 | 0 | 0 | 2 | 0 | 2 | 0 | 1 | 11  (30.56%) |
| Jin 2021 | 1 | 0 | 0 | 1 | 3 | 1 | 1 | 0 | 2 | 2 | 7 | 3 | 0 | 0 | 0 | 3 | 24  (66.67%) |
| Lee 2021 | 1 | 1 | 0 | 0 | 3 | 0 | 0 | 0 | 1 | 0 | 0 | 2 | 0 | 0 | 0 | 1 | 9  (25%) |
| Liu 2017 | 1 | 0 | 0 | 1 | 3 | 1 | 0 | 1 | 2 | 2 | 0 | 2 | 0 | 2 | 0 | 2 | 17  (47.22%) |
| Lizhi 2020 | 1 | 1 | 0 | 0 | 3 | 1 | 0 | 0 | 2 | 0 | 0 | 5 | 0 | 0 | 0 | 1 | 14  (38.89%) |
| Ma 2024 | 1 | 1 | 0 | 0 | 3 | 0 | 0 | 0 | 1 | 0 | 0 | 2 | 0 | 2 | 0 | 1 | 11  (30.56%) |
| Nardone 2022 | 2 | 1 | 0 | 1 | 3 | 0 | 0 | 1 | 1 | 0 | 0 | 4 | 0 | 0 | 0 | 1 | 14  (38.89%) |
| Natally 2018 | 1 | 0 | 0 | 0 | 3 | 0 | 0 | 0 | 2 | 0 | 0 | 2 | 0 | 0 | 0 | 1 | 9  (25%) |
| Pang 2021 | 1 | 1 | 0 | 0 | 3 | 0 | 0 | 0 | 2 | 0 | 0 | 3 | 2 | 0 | 0 | 1 | 13  (36.11%) |
| Peng 2023 | 1 | 1 | 0 | 1 | 3 | 1 | 0 | 1 | 2 | 0 | 0 | 2 | 2 | 0 | 0 | 1 | 15  (41.67%) |
| Peng^a^ 2023 | 1 | 1 | 0 | 1 | 3 | 1 | 0 | 1 | 2 | 0 | 0 | 2 | 0 | 0 | 0 | 1 | 13  (36.11%) |
| Ricardo 2019 | 1 | 0 | 0 | 1 | 3 | 0 | 0 | 0 | 1 | 0 | 0 | 2 | 0 | 2 | 0 | 1 | 11  (30.56%) |
| Shi 2023 | 1 | 1 | 0 | 0 | 3 | 1 | 0 | 1 | 2 | 0 | 7 | 4 | 0 | 0 | 0 | 2 | 22  (61.11%) |
| Wan 2021 | 1 | 1 | 0 | 1 | 3 | 0 | 0 | 0 | 2 | 0 | 0 | 2 | 0 | 0 | 0 | 1 | 11  (30.56%) |
| Wei 2023 | 1 | 0 | 0 | 1 | 3 | 1 | 0 | 0 | 2 | 0 | 0 | 3 | 0 | 0 | 0 | 1 | 12  (33.33%) |
| Wen 2023 | 1 | 1 | 0 | 1 | 3 | 1 | 0 | 0 | 1 | 1 | 0 | 2 | 0 | 2 | 0 | 1 | 14  (38.89%) |
| Yanfen 2018 | 1 | 1 | 0 | 0 | 3 | 1 | 1 | 0 | 2 | 2 | 0 | 2 | 0 | 2 | 0 | 1 | 16  (44.44%) |
| Yardimci 2023 | 1 | 1 | 0 | 0 | 3 | 1 | 0 | 0 | 2 | 0 | 0 | 2 | 0 | 2 | 0 | 1 | 13  (36.11%) |
| Yi 2019 | 0 | 1 | 0 | 0 | 3 | 1 | 0 | 0 | 1 | 0 | 0 | 2 | 0 | 0 | 0 | 1 | 9  (25%) |
| Zhang 2020 | 0 | 1 | 0 | 1 | 3 | 0 | 0 | 0 | 2 | 0 | 7 | 2 | 0 | 2 | 0 | 2 | 20  (55.56%) |
| Zhang 2022 | 1 | 1 | 0 | 1 | 3 | 0 | 0 | 0 | 2 | 0 | 0 | 2 | 0 | 2 | 0 | 1 | 13  (36.11%) |
| Zhihui 2021 | 1 | 1 | 0 | 0 | 3 | 1 | 1 | 0 | 2 | 1 | 0 | 3 | 0 | 2 | 0 | 1 | 16  (44.44%) |
